# From water to land—Usage of Generalized Unified Threshold models of Survival (GUTS) in an above-ground terrestrial context exemplified by honeybee survival data

**DOI:** 10.1093/etojnl/vgae058

**Published:** 2025-01-06

**Authors:** Leonhard Urs Bürger, Andreas Focks

**Affiliations:** Osnabrück University, Osnabrück, Lower Saxony, Germany; Osnabrück University, Osnabrück, Lower Saxony, Germany

**Keywords:** dose-response modeling, toxicokinetics, toxicodynamics, terrestrial invertebrate toxicology, pesticides

## Abstract

In regulatory aquatic risk assessment, toxicokinetic-toxicodynamic (TKTD) methods, such as the generalized unified threshold model of survival (GUTS), are already established and considered ready for use, whereas TKTD methods for aboveground terrestrial species, like arthropods, are less developed and currently not intended for risk assessment. This could be due to the fact that exposure in aboveground terrestrial systems is more event-based (feeding, contact, overspray, etc.), whereas exposure in aquatic systems is simply related to substance concentrations in the surrounding water. To provide a generic TKTD framework for terrestrial invertebrates, we propose a new GUTS variant that includes an intermediate buffer between the external exposure and inside of the organism. This buffer can be interpreted as residues on the exoskeleton or in the stomach, depending on the uptake route. Such an uptake behavior is mechanistically reasonable and observable in laboratory experiments. This GUTS variant, BufferGUTS, is particularly suitable for discrete or discretized exposure scenarios. Testing our model on honeybee datasets for 13 pesticides reveals a similar or better reproduction of survival curves than existing models (GUTS-RED and BeeGUTS) while keeping the number of parameters the same and making no substance or species-specific assumptions. The proposed new BufferGUTS approach can prospectively be used to derive TKTD parameters for a variety of terrestrial arthropod species. A standardized model definition for terrestrial species will facilitate the comparison and extrapolation of parameters between species and the applicability for terrestrial risk assessments.

## Introduction

Conducting risk assessments for a wide range of substances and species traditionally requires numerous laboratory tests involving animals. To reduce the need for such tests, new approach methods (NAMs), including in silico computer-based models, can be used in environmental risk assessment (Di Nicola et al., 2023). Among these, toxicokinetic-toxicodynamic (TKTD) models are particularly useful as they mechanistically simulate the uptake and effects of substances in organisms (Ashauer & Escher, 2010). Depending on the available data and the modeling objectives, different TKTD models may be suitable. For survival predictions based on exposure concentrations, general unified threshold model of survival models (GUTS; Jager et al., 2011; Jager & Ashauer, 2017) are most commonly utilized and are ready for application in aquatic environmental risk assessments (European Food Safety Authority (EFSA), 2018).

So far, TKTD models have primarily been used for aquatic (Ashauer et al., 2013; Jager et al., 2011; Rubach et al., 2010) or soil-dwelling species (Bart et al., 2023; van Ommen Kloeke et al., 2012), which are continuously exposed by their surrounding medium or a constantly contaminated food source (Jager, 2020). In aboveground terrestrial scenarios, however, exposure is less mediated by the surrounding medium (air), and is also not constant, but event-related. These events relevant to arthropods include overspray from agricultural practices, feeding on exposed plants or organisms, or contact with exposed surfaces like soil or plants. In contrast to relatively homogeneous water and soil systems, aboveground environments exhibit a highly fragmented exposure mosaic (Sponsler et al., 2019). Therefore, specific experimental systems that test for particular uptake routes and their effects are currently used in risk assessment, with exposure varying between routes. For example, oral tests use dietary concentrations (Organisation for Economic Co-operation and Development [OECD], 1998a, 2017), topical applications use droplet concentrations (OECD, 1998b), and glass-plate contact tests use substance amounts per area (Candolfi et al., 2000). The harmonization of exposure from these different tests into one common unit relevant for all routes in a terrestrial context can therefore be challenging. Caused by different and more complex uptake routes, exposure concentrations that are required to induce a certain effect on survival may differ more between different compounds than observed in aquatic systems. This can lead to high correlations between TKTD parameters and effect concentrations and can complicate comparisons of TKTD model parameters that aim for a deepened mechanistic understanding. Such comparisons can reveal differences in the uptake of chemicals that lead to variation in internal concentrations and thus in damage to the organism (Prado et al., 2020; Vidkjær et al., 2021). In particular, the absorption rate of topically applied chemicals depends to a large extent on the physicochemical properties of the pesticide and solvent used and their effect on the exoskeleton penetration ability of the compounds (Zaworra et al., 2019).

The only integrative TKTD modelling approach that combines different uptake routes so far was introduced by Baas et al. (2022) for honeybees, called BeeGUTS. It combines uptake routes by preprocessing exposure events via different routes into a common exposure proxy based on physiological and substance information. Substance amounts being applied to or consumed by each individuum, in the following called body dosages, are used as common unit, which works well for acute exposures. For chronic exposures, this conversion can lead to problems, because body dosages do not consider degradation or excretion. In consequence, this might suggest that lower body dosages cause higher mortalities for some datasets (see Figure 2 in Baas et al., 2022).

The main aim of this study is to improve TKTD models and workflows for a wide range of aboveground species such as arthropods. For this purpose, we propose a robust and generalized approach that relates event-based exposure to survival effects without relying on physiological knowledge in a conceptually sound way. Such physiological information is not readily available for many species and cannot be easily applied to a wide range of substances. Specifically, we suggest to discretize exposure events and combine this with a new GUTS model variant (BufferGUTS) that uses an intermediate buffer between the external exposure and inside of the organism. This buffer can be interpreted to represent residues on the exoskeleton or in the stomach, depending on the uptake route. A secondary aim of this study is to facilitate the comparability of modeling results across these routes and substances by normalizing exposure concentrations. For this purpose, we suggest using toxic units (TUs) as a common unit that is independent of specific routes and thus valid for all routes. We are aware that TUs seem to be a regression in time, because they depend on effect thresholds that depend again on specific observation times. The analyses in this study show, however, that the proposed methodology allows a more meaningful comparison of TKTD parameters for a wide range of chemical compounds across different exposure routes.

## Materials and methods

### Bee data

The dataset used in this study was derived from 38 standard regulatory reports concerning 13 different pesticide compounds for the honey bee *Apis mellifera*. These reports were selected based on the studies utilized in the BeeGUTS analysis (Baas et al., 2022) and were available through the Bayer transparency initiative (1994, 2019; reports used are listed in online supplementary material Table S4. As part of the transparency policy of Bayer Crop Sciences, the full reports can be requested by sending an email to cropscience-transparency@bayer.com referring to the report numbers listed in the supporting information). To ensure the reliability of the data, only studies with at least one treatment replicate above 30% mortality were included in the analysis. The final dataset comprised 51 exposure and survival datasets, including 1,070 biological replicates (controls included) in total for 13 different pesticides across 284 noncontrol exposure levels via three different exposure routes. Each biological replicate represented one batch of bees, with each concentration level being replicated a minimum of three times. In acute oral (OECD No. 213; OECD, 1998a) experiments, totaling 24 datasets, contaminated sugar with varying chemical concentrations was offered for up to 6 hr. In chronic oral (OECD No. 245; OECD, 2017) exposures, represented by eight datasets, contaminated food was offered for up to 10 days. In acute contact (OECD No. 214; OECD, 1998b) tests, totaling 19 datasets, dissolved doses of the test compounds were applied directly to the thorax of the bees. Bees were observed in groups of 10, with survival recorded at least daily for periods ranging from 2 to 10 days. Acute datasets were processed for modeling by determining the active chemical doses per bee (e.g., μg/bee), which were derived from the dietary exposure measured for each group of 10 bees or from the compound application directly to each bee. In the case of chronic tests, the exposure was represented by the levels of food contamination (e.g., mg/kg food).

### Current TKTD framework

In this study, due to the absence of measured body residues in standard ecotoxicological tests, reduced GUTS (GUTS-RED) models were utilized (Jager et al., 2011; Jager & Ashauer, 2017). These models link an external chemical concentration C to an internal scaled damage D using a dominant rate constant $k_{d}$, and they further connect this damage D to survival S via specific death mechanisms. The two primary GUTS death mechanisms are stochastic death (SD) and individual tolerance (IT). In the SD mechanism, survival is unaffected until a damage threshold z is exceeded, beyond which a killing rate d is applied, leading to stochastic mortality. The IT mechanism, on the other hand, assumes that effect thresholds z are distributed according to a log-logistic distribution with a median α and shape parameter β and when an individual’s tolerance threshold is surpassed, it dies instantly. Additionally, all GUTS models incorporate a background mortality parameter $h_{b}$ to account for mortality unrelated to exposure, modeled as a first-order decline.

In the BeeGUTS model, both GUTS-RED versions are combined with preprocessed exposure that are calculated based on specific physiological bee and substance properties. For acute contact exposure, Baas et al. (2022) assume a first-order decline with a rate of 0.4 d^−1^. For acute oral exposures, a constant increase during the feeding period is followed by a first-order decline with a rate of 0.625 d^−1^. Chronic exposure is treated as constant over the entire exposure period. The model uses as exposure unit, body dosage in μg/bee, for all pathways and feeds resulting effective concentrations as dose metric into the GUTS-RED models. This exposure preprocessing approach is specific to each exposure type and has to be adapted for each species. Currently, parameterizations are available for the honey bee and five other bee species (Baas et al., 2024). We use BeeGUTS to benchmark our results, because BeeGUTS is currently the only model that can describe results of different standard bee tests that act via different routes of exposure within one framework.

### Exposure data to exposure profile

**Figure 1.**
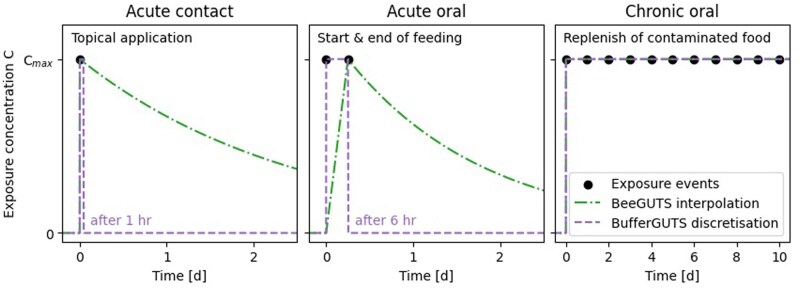
Interpolation of discrete concentration datapoints using BeeGUTS assumptions and an hourly discretization used for the BufferGUTS model. Exposure events include concentration C_max_ and timepoint of applied dosage (acute contact), concentration C_max_ and start and end timepoint of feeding (acute oral), replenish with exposure concentration C_max_ of contaminated food source (chronic oral).

**Figure 2.**
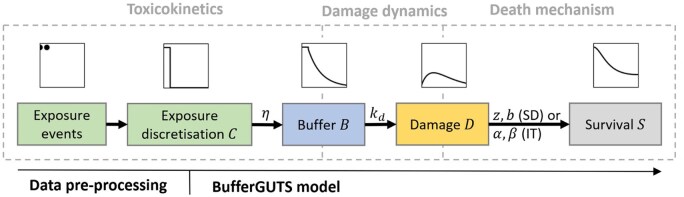
BufferGUTS model outline including the pre-processing of exposure events. The plots show exemplary time courses of the different state variables for a BufferGUTS-SD parametrization. SD = stochastic death.

To translate exposure events such as spraying or feeding into exposure profiles that are suitable for dynamic modeling, we propose a generic method that is independent of specific species properties or exposure pathways. Chronic exposure, such as long-term contamination of surfaces or food sources, can be modeled as a constant exposure throughout the modeling period. However, acute and event-driven exposures, like overspray events or consumption of recently contaminated matter, are more difficult to handle. To address this, we propose to discretize the exposure by assuming a constant level during the exposure period and zero exposure otherwise. The exposure period is in this case the time period where exposure events happen. These events include the moment of topical application, the start and end of the feeding period or the replenishing of the contaminated food resource (see Figure 1). For other datasets, it could also be the moment when individual organisms enter or leave a contaminated surface or any other kind of exposure that can be expressed as an event. It should be noted, that this step of exposure discretization is only a preprocessing step that does not claim to capture reality in the sense that the exposure falls down to zero after 1 hr, but it only fuels the uptake of a substance that is handled in the next compartment, the buffer. The BufferGUTS approach separates in this sense the contact time between the organism and the contaminated environmental compartment from the dynamics of uptake (Figure 2). In contrast, the BeeGUTS approach includes both elements in one (preprocessing) step by using species-specific physiological data (see Figure 1).

To ensure that the discretization step results in an area under the curve for all exposure profiles, we discretized the exposure data using a smallest time unit, which is set to 1 hr for this study. Consequently, (quasi-) instant exposures, like overspray events, are assumed to be occurring over 1 hr. We tested different smallest time units and found only minor impacts on the model outcome (see online supplementary material Figure S1). Feeding events that can last for several hours are discretized in the same way, maintaining a constant level during the feeding period and zero afterward. Therefore, acute contact datasets are represented as constant for 1 hr and zero thereafter, whereas acute oral datasets are constant during the feeding period of up to 6 hr and zero afterwards. Chronic oral datasets maintain a constant exposure throughout the entire test period.

### BufferGUTS

Although GUTS-RED models are generally capable of using discretized external exposure profiles as input, we propose a new GUTS model variant that includes an additional buffer compartment B. This buffer compartment is used to account for a delay between exposure events and the uptake into an organism. Depending on the exposure route, it might be interpreted, for example, as mass in the stomach or on the exoskeleton, so a compartment that has to be passed by any substance before affecting the organism. The addition of a buffer allows consideration of delayed effects on survival for acute exposures, like short-time feeding on contaminated food, overspray of terrestrial arthropods, or presence on a contaminated surface. The concentration in the buffer is thus the concentration that is relevant for damage accrual and its temporal behavior is similar to external substance residues from laboratory studies (Haas et al., 2021; Zaworra et al., 2019). In the model, the buffer B is filled (quasi) immediately with the external exposure concentration C and declines with first-order kinetics if the external concentration C falls below the concentration in the buffer. Unlike the BeeGUTS model, this first-order kinetic is directly linked to the scaled damage D accrual by using the same dominant rate constant $k_{d}$ for both processes. The scaled damage D is then used for the IT or SD death mechanism in the standard way of reduced GUTS models. A schematic overview of the model is shown in Figure 2.


(1) if: B(t)≤C(t)  Bt≔C(t)  else: dBtdt=kd(C(t)-B(t))

(2) dD(t)dt=kdB(t)-D(t)

In this proposed model, the buffer B replaces the (internal) concentration values used in other GUTS models and can be derived from the full GUTS model (Jager & Ashauer, 2017; see the online supplementary material for a description). Using the dominant rate constant $k_{d}$ for buffer elimination and damage accrual assumes that both processes are directly connected and for cases with no external concentration C and no damage D, elimination and accrual have the same absolute value, that is, the same process dominates the kinetics. In terms of model complexity, this “dual use” enables the dominant rate constant $k_{d}$ to govern the shape and temporal behavior of the scaled damage D in relation to the exposure concentration C (see online supplementary material Figure S3 for examples) to keep the number of parameters low. A comparable inverse correlation between concentrations on the exoskeleton and the accrual of internal concentrations was also observed in laboratory experiments (Haas et al., 2021; Zaworra et al., 2019). It has to be noted that BufferGUTS follows the reduced GUTS approach in the sense that it does not aim at modeling internal concentrations explicitly, but it uses scaled state variables in the same unit as the external exposure concentration instead. This approach simplifies the units of state variables and parameters, which relate only to time and/or the external substance concentration (see online supplementary material Table S2 for all units).

### TU conversion

Using the same time and external concentration unit across all datasets is beneficial to base all parameters on the same units for later comparisons. Time in GUTS models is generally reported in days and therefore used here as well (e.g., Jager et al., 2011). Finding a common unit for all external concentrations is more challenging because the toxicity of the various compounds tested in this study shows substantial variability, with lethal doses (LD50s) ranging from nanograms per bee to milligrams per bee. Using such an absolute unit as the common exposure unit results in a wide range of parameter values for the different compounds. Although this is not inherently problematic, some resulting parameter values will be highly correlated to the LD50 (e.g., effect thresholds are always smaller than the LD50). To avoid such a correlation in the parameter values, we normalized all exposure concentrations using the TU. Toxic units are calculated by dividing all exposure concentrations from one study by their respective lethal concentration (LC50) or LD50 values, as observed at 48 hr in acute tests or at 10 days in chronic tests. This normalization aims at avoiding problems with high interstudy toxicity variability and is calculated using a logistic fit $h\left(c\right)$ of the survival datapoints with concentrations $c_{i}$.


$$h\left(c_{i}\right)=\frac{1}{1+{\left(c_{i}/LC_{50}\right)}^{b}}$$

This conversion additionally facilitates parameter optimization by simplifying the definition of priors or starting values for optimization algorithms. Because the conversion to TU is a linear scaling of input exposure concentrations to ease parameter comparison and generalization, modeling results and parameter values can be scaled back to their original units using the LC50 or LD50 values (see online supplementary material for an example).

### Parameter optimization

Optimization of free model parameters was performed per each dataset using a Bayesian inference approach. The inference process is carried out using the state-of-the-art Markov chain Monte Carlo sampler NUTS developed by Hoffman & Gelman (2014), which is an implementation of the Metropolis-Hastings algorithm (Hastings, 1970; Metropolis et al., 1953) in the PyMC Python package (Ver. 5.10, Abril-Pla et al., 2023). The optimization process was set to use eight chains and consists of 5,000 tune and draw steps, aiming for a target acceptance rate of 0.8. Weakly informative priors are defined for each model individually using a preliminary fit with only 1,000 tune and draw steps, allowing for parameters to vary within the range of 10^−10^ to 10^3^. Model differential equations are solved using a forward Euler approach with a fixed step size (1/1,000 of the exposure time). To evaluate the optimization procedure, we tested our procedure with ringtest data from Jager & Ashauer (2017) against the OpenGUTS (openguts.info) and MORSE (Baudrot & Charles, 2021) implementations. All three implementations result in comparable optimal parameter values and confidence or credible intervals (see online supplementary material Table S3). Whereas the OpenGUTS implementation uses a frequentist Nelder-Mead approach for the parameter optimization, MORSE also uses a Bayesian Markov chain Monte Carlo approach based on the same Metropolis-Hastings algorithm. The main differences to our implementation are a different sampler (JAGS) and more restrictive parameter ranges in the optimization. Both samplers should behave comparably for GUTS problems and a wider allowable range helps to avoid running into parameter range limits.

Both the GUTS-RED and BufferGUTS models are optimized based on the discretized exposures as detailed above. For BeeGUTS model fits, GUTS-RED models are optimized using the BeeGUTS precomplied exposures as defined by the authors.

### Model performance metrics

In assessing the calibration performance of the models, we used the three criteria mentioned in the scientific opinion for model validation (EFSA, 2018). These include the predictive posterior check (PPC), the normalized root-mean-square-error (NRMSE) and the survival probability prediction error (SPPE, Focks et al., 2018). The PPC evaluates the agreement between the predicted number of survivors and their associated uncertainty limits with the observed number of survivors. The PPC values express the proportion of observed survivor numbers falling within the predicted uncertainty limits relative to all observations. A PPC value of 100% indicates that all observed data points lie within the uncertainty limits of the model predictions, whereas values below 50% indicate poor model performance. The NRMSE normalizes the classical root mean square error by the mean of all observations, delivering a criterion that can be compared across datasets. Perfect agreement between predicted and observed survival numbers was indicated by NRMSE values of 0%, whereas values below 50% are considered acceptable in the EFSA TKTD scientific opinion. The SPPE quantifies the difference between the observed and predicted survival numbers at the last timepoint of each treatment, normalized by the initial number of individuals. Each replicate is assigned an SPPE value, with 0% indicating perfect prediction, negative values suggesting underestimation, and positive values indicating overestimation of effects. The range of over and underestimation is illustrated by reporting the maximum and minimum SPPE values per dataset.

## Results and discussion

### Model performance

**Figure 3.**
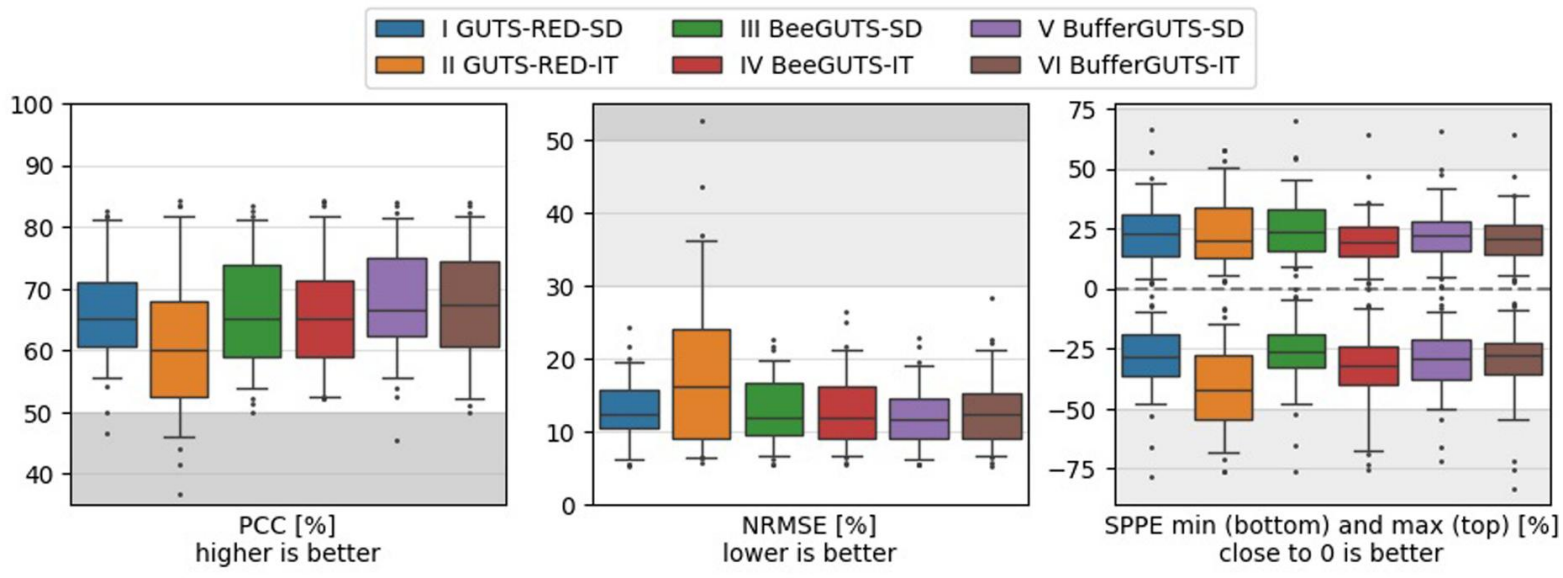
Model performance metrics predictive posterior check (PPC), normalized root mean square error (NRMSE) and survival probability prediction error (SPPE) over all 51 datasets as described by EFSA (2018). Dark-grey areas indicate a poor model performance according to EFSA (2018) and light-grey according to the BeeGUTS article (Baas et al., 2022). Whiskers show 5^th^ to 95^th^ percentiles. SD = stochastic death; IT = individual tolerance.

The results for the model performance metrics, as defined in the EFSA Scientific Opinion (EFSA, 2018), in Figure 3 demonstrate generally acceptable performance across all tested models with the exception of the GUTS-RED-IT model, which exhibits notably poorer performance compared with the others (see online supplementary material Figures S4–S6 for individual study results). The majority of models achieve a PPC passing rate of more than 60% across most datasets, maintain a NRMSE below 20%, and display SPPE minimum/maximum values ranging approximately between 20% to 30%. This suggests that most models allow for a successful optimization of the four model parameters to establish a linkage between exposure concentrations and observed survival outcomes. However, it is important to note that the mechanisms through which different models mechanistically establish this linkage vary considerably.

**Figure 4.**
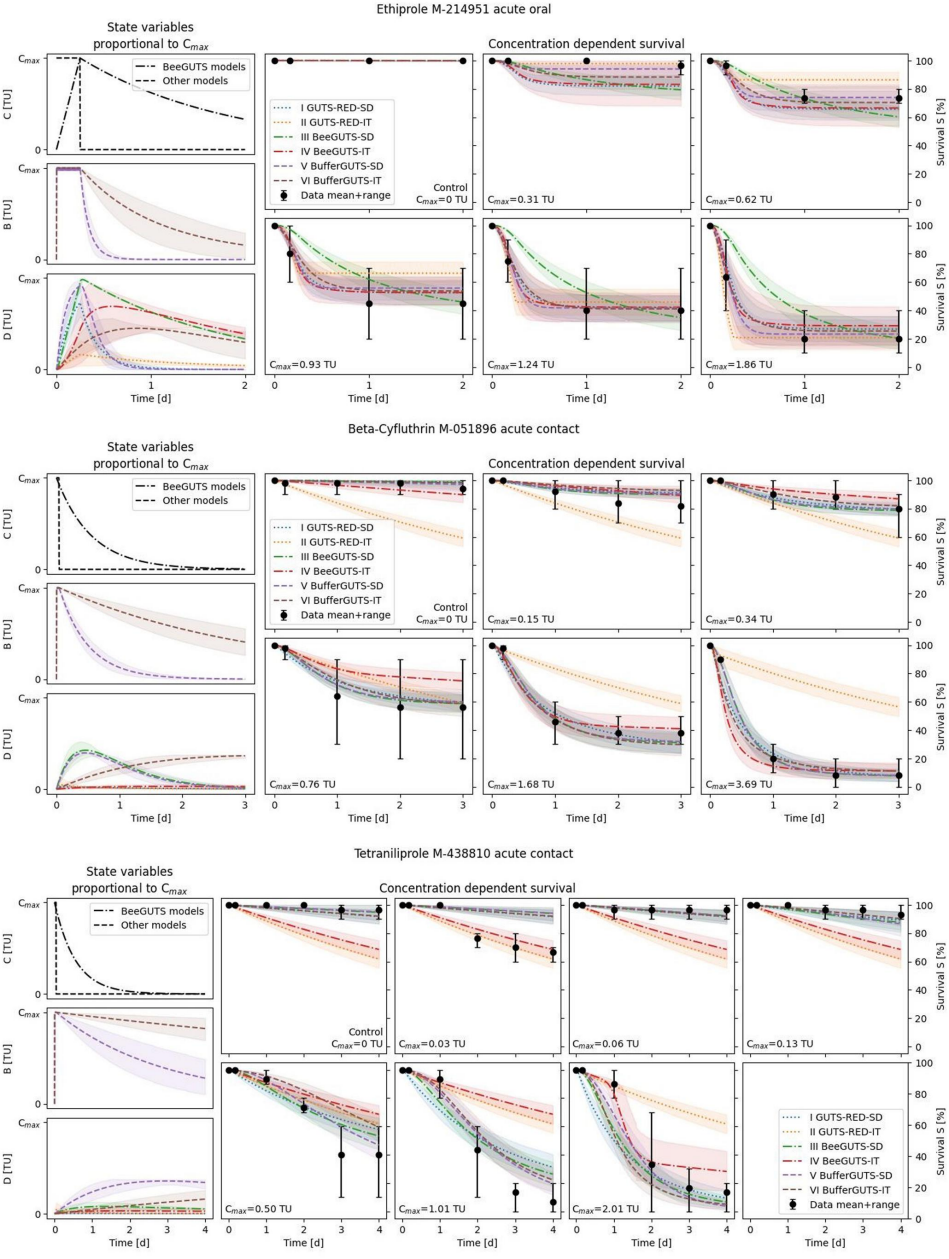
Calibration results of the different generalized unified threshold model of survival (GUTS) variants tested for example datasets showing fast kinetics (top, ethiprole), medium kinetics (middle, beta-cyfluthrin) and slow kinetics (bottom, tetraniliprole). The state variables exposure concentration C, buffer B and damage D are all expressed in toxic units (TU). SD = stochastic death; IT = individual tolerance.

Figure 4 presents three example datasets for different chemicals, representing cases with fast (Figure 4 top, ethiprole), medium (Figure 4 middle, beta-cyfluthrin) and slow kinetics (Figure 4 bottom, tetraniliprole). In the case of fast kinetics, mortality already occurs during the acute feeding phase of 6 hr. Here, the BeeGUTS-SD model encounters challenges that are related to the used exposure preprocessing. The linear increase of exposure as resulting from the preprocessing, coupled with the SD death mechanism that is related to the current damage, inhibits early mortality in the model, whereas the fixed first-order exposure decline results in elevated mortality at later observation times. For the medium kinetic case, most deaths occur between the 6-hr and 24-hr timepoints, leading to a dominant rate constant $k_{d}$ (0.3 to 0.7 d^−1^) of the BufferGUTS-SD model close to the BeeGUTS first-order decline parameters (0.4 d^−1^ and 0.625 d^−1^). This results in very comparable damage and survival kinetics between BeeGUTS and BufferGUTS models for these substances. However, the GUTS-RED-IT model struggles to fit such data due to its inability to predict mortality beyond the exposure phase due to its death mechanic that depends on the maximum damage. Here, increased background mortality rate constants $h_{b}$ try to capture the mortality the calibration of the GUTS-RED-IT model. For the slow kinetics case, where mortality occurs gradually over multiple days, the GUTS-RED-IT and BeeGUTS-IT models encounter difficulties. The BeeGUTS preprocessed exposure is already close to zero here, although mortality still occurs, leading to the same problem as observed for IT models that rely on the maximum damage. BufferGUTS models and GUTS-RED-SD, on the other hand, demonstrate the ability to reproduce survival patterns in all three cases.

When scaled damages D are additionally taken into consideration, GUTS-RED-SD stands out concerning the estimated parameter values. It is often calibrated with very small dominant rate constants $k_{d}$ to create an almost linear increase in damage D in combination with very small thresholds z and high killing rates b values to capture the mortality. Although this approach effectively reproduces most survival patterns, it lacks mechanistic plausibility. In contrast, BufferGUTS models have scaled damages D in the range of the exposure concentration $C_{\max}$ across all kinetic speeds, indicating a mechanistically plausible reproduction of the process. Detailed fits for all 51 datasets are provided in the online supplementary material.

### Parameter values and credibility intervals

**Figure 5.**
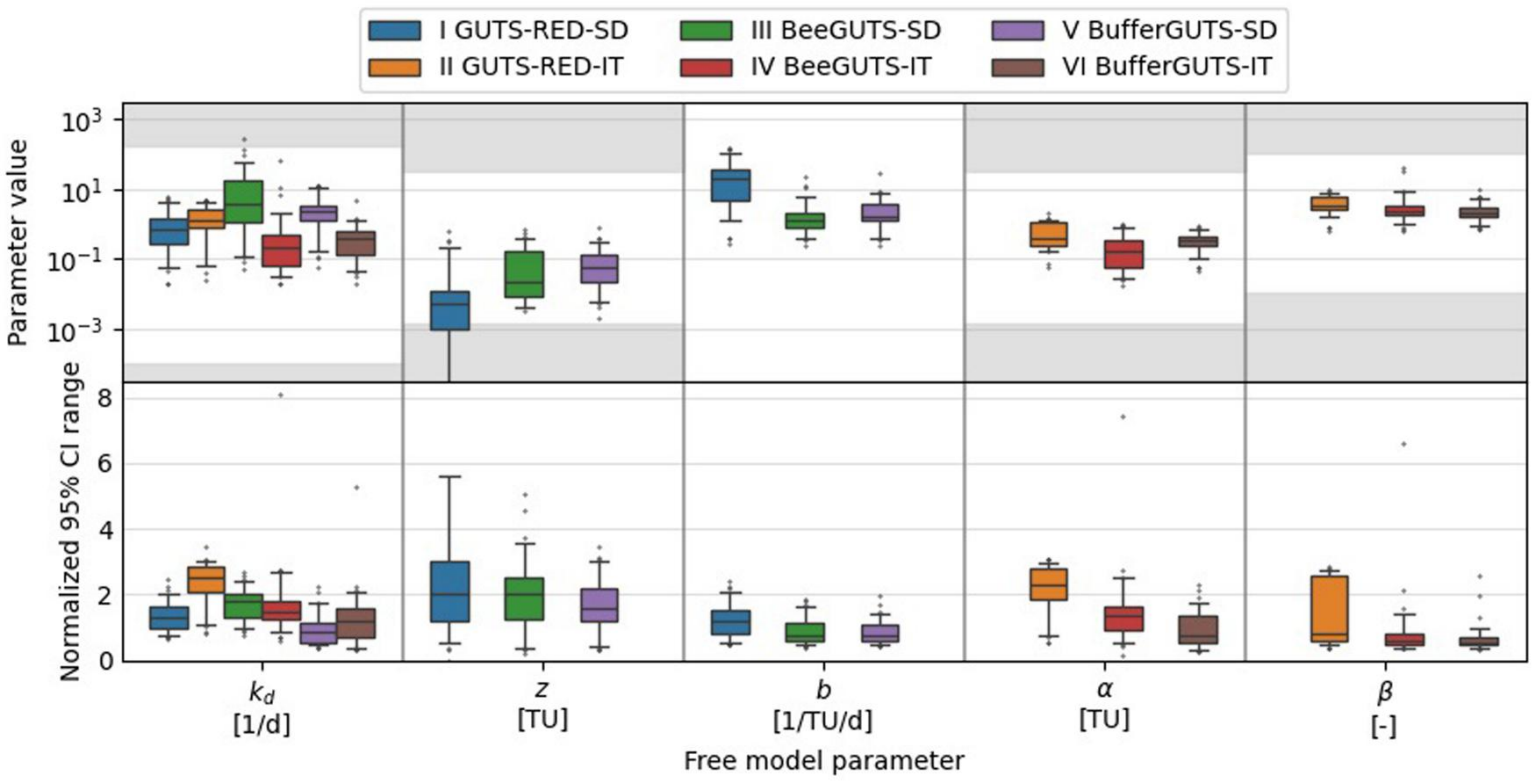
Calibrated parameter values (top) and 95% credibility interval (CI) ranges normalized by the parameter value (bottom) for all tested models and 51 datasets. Some parameters are given in toxic units (TU) and background mortality h_b_ values are not shown. Grey areas are unlikely parameter ranges based on Delignette-Muller et al. (2017) derived for all datasets. Whiskers show 5^th^ to 95^th^ percentiles, the whisker for the threshold *z* of the GUTS-RED-SD model goes down to the minimum parameter value of 10^−10^. SD = stochastic death; IT = individual tolerance.

Parameter values generally fall within similar ranges across all models (Figure 5; also see online supplementary material Figures S7–S9 for results sorted by different uptake routes) because external concentrations are expressed in TU. However, the GUTS-RED-SD model stands out with notably small effect threshold z values and high killing rates b. The consistency in parameter values across models enhances their absolute relevance and facilitates potential extrapolation across species and chemicals (van den Berg et al., 2019; van den Berg et al., 2021). Notably, parameters such as the killing rate b and shape parameter β appear relatively constant across different compounds and uptake routes, suggesting the possibility of fixing them for BeeGUTS and BufferGUTS models for exposures expressed in TU (as seen in Singer et al., 2023). This further suggests an inverse relationship of the LD50/LC50 and the killing rate. Conversely, the dominant rate constant $k_{d}$ and threshold z or median α seem to depend more on the specific compound and uptake route (to compare, see online supplementary material Figures S7–S9). This can be explained, for example, by different uptake and effect speeds and suggests different sensitivities towards exposure from different routes. Parameters also exhibit comparable ranges across models and datasets, with the GUTS-RED models showing the widest credible interval (CI) ranges. A narrower CI range indicates more credible optimized parameter values and thus more reliable fits.

## Discussion

### Conceptual consistency and integration capacity of BufferGUTS

The BufferGUTS models conceptionally link external concentrations and damage via different uptake routes as demonstrated for experimental data from honey bees for different substances (Haas et al., 2021; Zaworra et al., 2019). The usage of an intermediate buffer state enables the model to adapt to substances with both rapid and slow kinetics. In contrast, the use of a fixed decline rate for each substance (like in BeeGUTS) can lead to problems in acute scenarios where mortality occurs already early during the exposure period or where the majority of mortality happens days after exposure (see Figure 4). Integrating event-based discretization with the BufferGUTS model provides a solid framework for an effective application of TKTD models to evaluate the impact of event-based aboveground terrestrial exposure on the survival without using specific physiological properties. This approach might be particularly useful for nontarget arthropods such as bees, spiders, and beetles.

The event-based exposure discretization can be effectively applied to model scales, whether using TKTD alone or in combination with exposure (e.g., Lonsdorf et al., 2024) and/or landscape models (e.g., Becher et al., 2014; Topping et al., 2003) that mostly operate on daily timesteps. Some pesticide-effect models have been combined with spatiotemporal exposure for some species, like freshwater crustaceans (van den Brink et al., 2007), earthworms (Roeben et al., 2020) or even bee colonies (Preuss et al., 2022). However, to our knowledge no TKTD module for an aboveground species with uptake and effect in combination with individual exposure based on, e.g., movement in a landscape model is currently available. Our discretization approach can be advantageous in such future modeling cases because it does not require additional assumptions about the modeled species or other components. Combining TKTD models with individual spatiotemporal exposure patterns could be done in the future by discretizing individual exposure patterns resulting from the landscape model based on the time unit used by the model and feeding them into BufferGUTS. The GUTS parameters should then be estimated from laboratory experiments using the same minimal time unit as the landscape model (e.g., one day). Such combination would allow to extrapolate effects of more realistic exposure scenarios from landscape models and could be validated using multi-exposure semi-field experiments.

### Using TU as exposure unit

Normalization of exposure was shown in this study to allow for a better comparison between different compounds and exposure routes. Without normalization, most parameter values depend on the absolute units of the exposure concentrations (e.g., 100 ng/bee or 10 µg/bee) derived from laboratory testing and cannot be directly compared. Shifting all exposures to a common unit (e.g., µg/bee) results in parameter values with many leading or trailing zeros due to differences in species sensitivity to the compounds being compared. Such differences are already visible from the LC50 or LD50 alone and do not require a TKTD model fit. This observation is true for all GUTS models and is only of minor importance when only one species, compound, and uptake route is investigated; in all other cases, normalization can be beneficial. Normalizing exposures and thus parameters to TU can give additional insight in the behavior of GUTS models and the toxicity of compounds. Our results based on normalized exposure in units of TU show, on the one hand, the killing rate b to be basically independent of other substance properties and the uptake route, making it only depended on the substance LC50 or LD50. The threshold *z*, on the other hand, appears to also depend on other substance properties and the uptake route (see online supplementary material Figures S7–S9). Having parameters in a normalized unit like TUs makes this finding more obvious by removing the correlation to the substance toxicity. In absolute units, it would be hard to realize, for example, that some substances show an effect at less than 1% of their LC50 whereas others have their threshold z above 10% of their LC50 (see Figure 5 BeeGUTS and BufferGUTS models).

Finding a common unit that is meaningful across different uptake routes can be particularly challenging in context of terrestrial ecosystems. Each uptake route has its own exposure unit that depends on the source of contamination and exposure. For oral tests, it is the substance concentration in the food solution (OECD, 1998a, 2017) and for topical contact tests, the substance concentration in the applied droplets (OECD, 1998b). These units can be converted to absolute substance amounts per individuum, the here-called body dosage. In the BeeGUTS article, such body dosages are used to unify different routes, which work well for acute exposures but can lead to problems for some chronic oral datasets. The addition of the consumed compound quantities over the entire exposure period to a body dosage does not account for degradation or excretion and can lead to problematic interpretations when most individuals die early in chronic tests. In such cases, bees that consumed food with higher contamination and died early have lower body dosages than those surviving the whole test period. Consequently, working with body dosages might misleadingly suggest that lower exposure concentrations cause higher mortalities (see Figure 2 in Baas et al., 2022). These problems can be avoided, either by excluding treatment concentrations leading to all individuals dying very early in the experiment, assuming the same feeding rate for all treatments, or by using the unconverted exposure directly (substance concentration in the food solution).

When nontopical contact tests should also be included in the analysis, conversion to body dosages is no longer an option based on information on the experimental setup alone. Such tests are, for example, used in arthropod risk assessment for *Aphidius rhopalosiphi* and *Typhlodromus pyri* (Candolfi et al., 2000). A conversion to TUs is still possible because an LC50 or LD50 can be calculated for all kinds of experimental designs and potential uptake routes. These values are timepoint specific and ideally the same timepoint is used for all uptake routes. Substantial differences in test designs and concentrations between acute and chronic tests can complicate this. Consequently, different timepoints are used in this study for acute (2 day) and chronic (10 day) datasets. Current test protocols could be adjusted for TKTD modeling with more monitoring events and a longer monitoring period (e.g., 10 days) to bridge this problem.

In this study, normalization was performed for each study individually to obtain ideal results for our model and parameter comparisons based on the individual studies. Another approach would be to normalize each combination of substance and uptake route individually. The resulting LC50 or LD50 for the substance and uptake route could then also be used to normalize new datasets with nonstandard exposures, like pulses.

Regardless of the normalization approach and timepoint used, it is important to note again that a normalization to TU only involves linear scaling of exposure and related parameters, similar to a unit shift. Thus, values can always be converted back to their original unit using the LC50 or LD50, maintaining consistency with other non-TU standardized datasets and analyses (see online supplementary material for an example). The BufferGUTS approach as such des not rely on using normalization to TUs.

### Validation criteria for calibration results

In our performance assessment, we applied model validation criteria to model calibration results; this means we did not fully follow the EFSA TKTD scientific opinion for aquatic species (EFSA, 2018). It needs to be discussed in the future whether the method of calibrating to constant data and validating on data with multiple pulses is transferable from aquatic to aboveground terrestrial species. In the BeeGUTS paper (Baas et al., 2022), models are calibrated using chronic feeding data and validated against single pulse acute datasets, assuming equal sensitivity across different uptake routes. A validation in line with the EFSA scientific opinion ideally would use datasets relevant for the exposure profiles that are to be expected for the environmental scenario in which the analyzed species live. For honeybees, for example, such a dataset could evaluate the impact of multiple pulsed exposures, which were not available to us. Because our model currently focuses on exposure routes individually, we could also not use the data from one exposure route to validate another, as done for BeeGUTS. Therefore, for this study, we relied on the findings of Bauer et al. (2024), showing that well-calibrated GUTS models also tend to perform well in validation.

Our main next goal is to expand BufferGUTS to cover different uptake routes for the same chemical into one conceptual model. Such a unification might take advantage of the relatively constant parameter values for the shape parameter β and killing rate b, suggesting that parameter sharing might be feasible. Additionally, an approach similar to Bart et al. (2021), addressing mixture effects of different compounds, could be used to account for sensitivity variations to exposures of the same compound via different uptake routes.

### Applicability to other species than honeybees

This study aimed at demonstrating that the model can effectively describe the effect dynamics per species, compound, and exposure route without using physiological knowledge and compared the results to BeeGUTS output as a benchmark. Because BufferGUTS does not include assumptions about species traits or exposure behavior, it can be assumed that it can calibrate to data from other terrestrial arthropods (for an example for a non-*Apis* bee; see online supplementary material Figure S10) and experimental designs as well without problems. A calibration across multiple routes could then be validated with independent pulsed data, thus ensuring robustness and reliability of the modeling.

Combining a normalization based on TUs with GUTS variants using consistent parameters enhances the comparability and transferability of parameters between species and compounds. Predicting LC50 and LD50 values for different aquatic species and compound combinations is already possible with high accuracy using quantitative structure–activity relationship (QSAR) approaches like Bio-QSAR (Zubrod et al., 2024). Similar approaches could be developed to predict GUTS parameters for other species or compounds in the future, helping to reduce the need for further animal testing in risk assessment.

## Supplementary Material

vgae058_Supplementary_Data

## Data Availability

As part of the transparency policy of Bayer Crop Sciences, the full reports can be requested by sending an email to cropscience-transparency@bayer.com referring to the report numbers listed in the supporting information.
