# Supplementary material for: From water to land—Usage of Generalized Unified Threshold models of Survival (GUTS) in an above-ground terrestrial context exemplified by honeybee survival data
Source: Environ Toxicol Chem. 2025 Jan 6;44(2):589–98. doi: 10.1093/etojnl/vgae058 (PMC11816312; doi:10.1093/etojnl/vgae058)
Supplement: vgae058_Supplementary_Data [file vgae058_supplementary_data.docx]

**From water to land – Usage of GUTS in an above-ground terrestrial context** **exemplified for honeybee survival data**

**Supporting Information**

Leonhard U. Bürger^1^ & Andreas Focks^1^

^1^Osnabrück University, Osnabrück, Germany

Leonhard.buerger@uni-osnabrueck.de

Table of content

[1. Model description 1](#_Toc177893228)

[2. Sensitivity analyses 3](#_Toc177893229)

[2.1 Different smallest time units for the exposure discretization 3](#_Toc177893230)

[2.2 Different dominant rate constants kd 3](#_Toc177893231)

[2.3 Different buffer speed $\eta$ values 4](#_Toc177893232)

[3. Toxic unit examples 4](#_Toc177893233)

[*4.* Example units for model parameters assuming different exposure units 5](#_Toc177893234)

[5. Additional figures and tables 6](#_Toc177893235)

[5.1 Model quality metrics 6](#_Toc177893236)

[5.2 Parameter values plotted per exposure route 7](#_Toc177893237)

[5.3 Model calibration benchmark results for GUTS ring-test data 9](#_Toc177893238)

[5.4 Calibration example for the non-Apis bee species Osmia bicornis 10](#_Toc177893239)

[5.5 Study Numbers of underlying toxicity data 11](#_Toc177893240)

[5.6 Complete calibration results 12](#_Toc177893241)

[6. References for the supporting information 29](#_Toc177893242)

# Model description

While GUTS-RED models are able to work with data discretised in such a way, we propose a new GUTS model using an additional buffer compartment $B$. This buffer compartment is used to account for a delayed damage accrual and thus effect on survival for acute non-water related exposures, like feeding or overspray of terrestrial arthropods. It should be as high as the exposure concentration $C$, as long as the exposure is higher than the buffer $B$ and decline with the dominant rate constant $k_{d}$ afterwards.

$$\begin{aligned} B(t)=C(t) if: B(t)\leq C(t) else: \frac{dB\left( t \right)}{dt}=k_{d}(C(t)-B(t))\#\left( 1 \right) \end{aligned}$$

The buffer $B$ thus replaces the (internal) concentration values of the other GUTS models and can be derived from the full GUTS model (Jager & Ashauer, 2017) by assuming an infinite uptake rate $k_{u}$ for the internal concentration and setting the elimination rate $k_{e}=k_{d}$ (Jager & Ashauer, 2017; p. 45; eq. 3.1) and the repair constant $k_{r}=k_{d}$ (Jager & Ashauer, 2017; p. 45; eq. 3.6). The (scaled) damage $D$ is thus related to the buffer $B$ via the dominant rate constant $k_{d}$.

Depending on the solver used, this formulation can pose a problem, because it is not a differential equation, thus an additional buffer speed constant $\eta\gg1$ can be introduced. It governs the speed with which the buffer is filled. It is not a fitable parameter and for $\eta\to\infty$ the differential equation version is equal to previous one. For solvers with a fixed integration step, as used here, $\eta$ should be set to fill the buffer within one integration step (here: 1/1000 of the exposure period). For solvers with variable integration steps an environmentally reasonable buffer speed $\eta$ leading to a filled buffer in e.g. seconds or minutes is sufficient when effects are expected be seen after a couple of hours or days. If the exposure and survival data is given in daily resolution, the buffer speed $\eta$ can be set to e.g. $86,400 1/d$ to fill the buffer within model seconds. Resulting buffer $B$ and scaled damage $D$ equations are thus:

$$\begin{aligned} \frac{dB\left( t \right)}{dt}=k(C(t)-B(t)) with k=\left\{ \begin{aligned} \eta, &B(t)\leq C(t) \\ k_{d}, &B(t)>C(t) \end{aligned} \right.\#(2a) \end{aligned}$$

$$\begin{aligned} \frac{dD(t)}{dt}=k_{d}\left( B(t)-D(t) \right)\#\left( 2b \right) \end{aligned}$$

Resulting damage $D$ can then be fed into either the SD or IT death mechanic of GUTS-RED (SI Table S1).

SI Table S1 Equations and parameters of GUTS-RED-SD and IT models from (Jager & Ashauer, 2017).

| GUTS-RED-SD | GUTS-RED-IT |
| --- | --- |
| $\begin{aligned} \frac{dD(t)}{dt}=k_{d}\left( C(t)-D(t) \right)\#\left( 3a \right) \end{aligned}$  $\begin{aligned} h_{z}=b\cdot max\left( 0,D(t)-z \right)+h_{b}\#\left( 3b \right) \end{aligned}$  $\begin{aligned} \frac{dS(t)}{dt}=-h_{z}S(t)\#\left( 3c \right) \end{aligned}$ | $\begin{aligned} \frac{dD(t)}{dt}=k_{d}\left( C(t)-D(t) \right)\#\left( 4a \right) \end{aligned}$  $\begin{aligned} D_{mw}=\max_{0<\tau<t} D\left( \tau\right)\#\left( 4b \right) \end{aligned}$  $\begin{aligned} S_{b}=exp\left( -h_{b}t \right)\#\left( 4c \right) \end{aligned}$  $\begin{aligned} S=S_{b}\int_{D_{mw}}^{\infty} f\left( z,\alpha,\beta\right)dz\#\left( 4d \right) \end{aligned}$ |
| $C$: External concentration  $D$: Scaled damage  $k_{d}$: Dominant rate constant  $z$: Threshold for effects | $b$: killing rate (just SD)  $f$: Log-logistic threshold distribution function (just IT)  $\alpha$: Median of $f$(just IT)  $\beta$: Shape parameter of $f$ (just IT) |

# Sensitivity analyses

## Different smallest time units for the exposure discretization


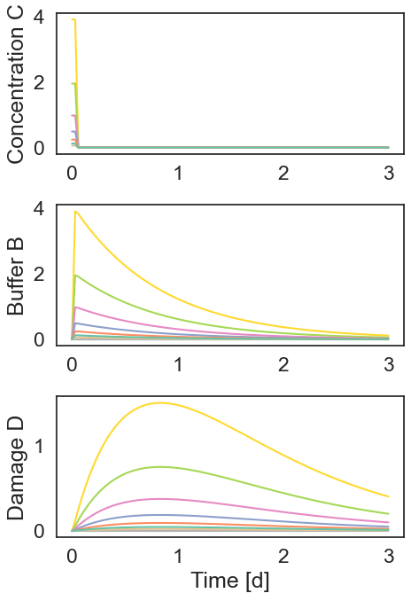

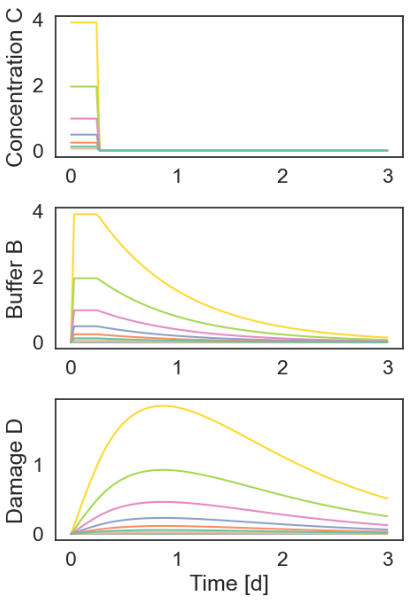

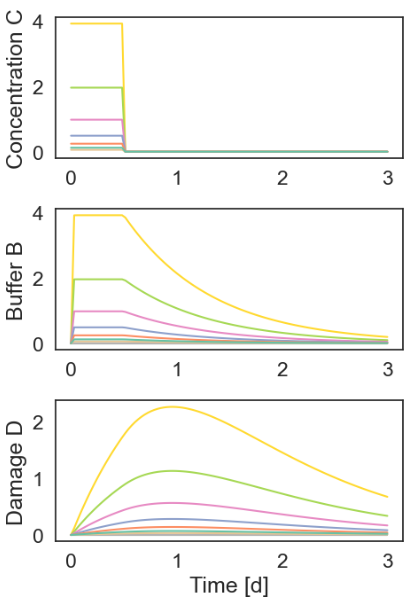


SI Figure S1 Buffer and damage dynamics with different smallest time units for the exposure discretization and all other parameters fixed ($\eta=1440 1/d$, $k_{d}=1.2 1/d$, concentration in TU). From left to right smallest time units of 1 hour, 6 hours and 12 hours. The time unit has only a minor influence on the shape of the damage, but its maximum value increases, due to a larger area under the exposure concentration curve.

## Different dominant rate constants kd


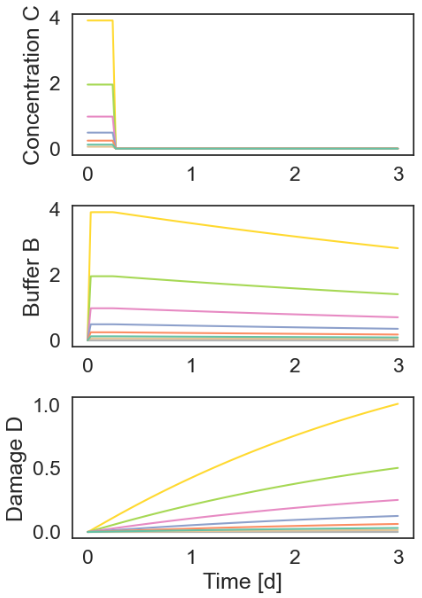

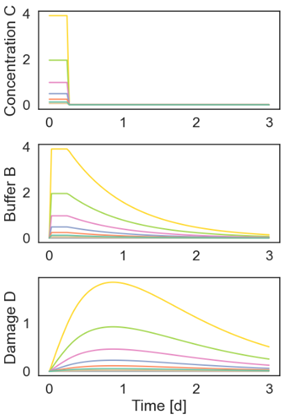

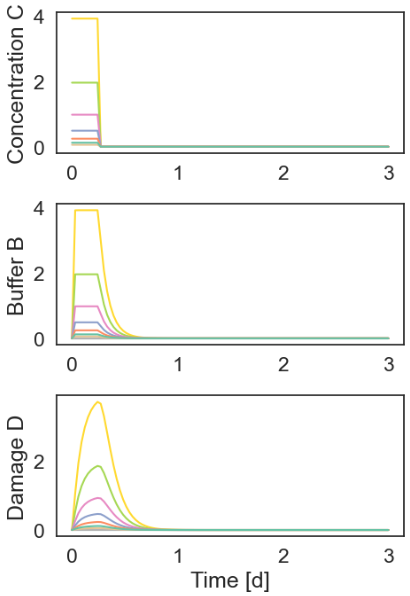


SI Figure S2 Buffer and damage dynamics with different dominant rate constants $k_{d}$ and all other parameters fixed ($\eta=1440 1/d$, concentration in TU). From left to right $k_{d}=0.12 1/d$ for slow kinetics,$k_{d}=1.2 1/d$ for intermediate kinetics and $k_{d}=12 1/d$ for fast kinetics.

## Different buffer speed $\eta$ values


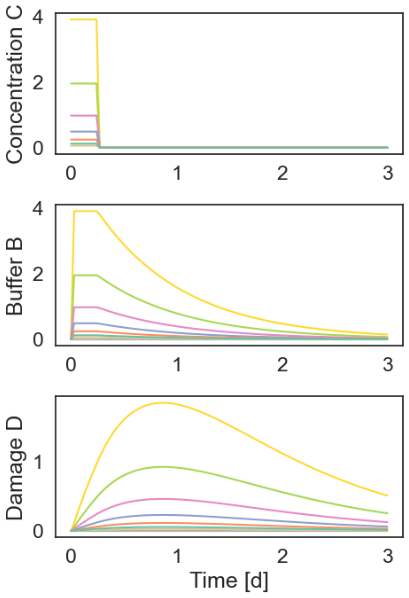

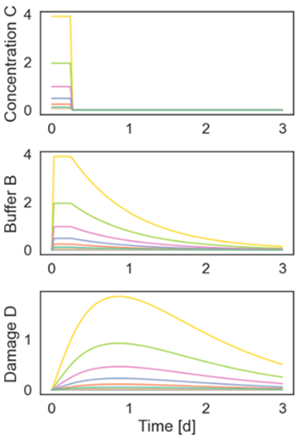

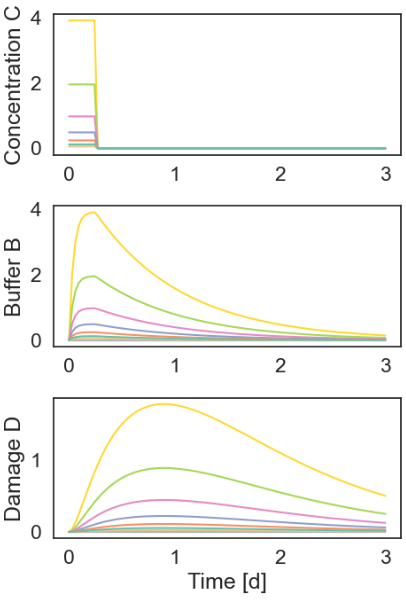


SI Figure S3 Buffer and damage dynamics with different buffer speed values $\eta$ and all other parameters fixed ($k_{d}=1.2 1/d$, concentration in TU). From left to right $\eta=86,400 1/d$ for a buffer speed within seconds, $\eta=1440 1/d$ for a buffer speed within minutes and $\eta= 24 1/d$ for a buffer speed within hours. Effect on the damage $D$ is negligible if the exposure duration is bigger than the time needed to saturate the buffer.

# Toxic unit examples

Toxic units (TU) derived by normalizing exposures with the LC50 or LD50 and resulting parameter fits can be easily transformed back to the original concentrations. As shown in the following example for a hypothetical compound with a LC50 of 200 mol/L, so 1TU = 200 mol/L. Thus, the TU conversion factor is 200 mol/L/TU.

An exposure concentration of 300 mol/L can then be transformed into TUs by:

$$\frac{300 mol/L}{200 mol/L/TU}=1.5 TU$$

A resulting fit for e.g. the GUTS-RED-SD model can have parameters $k_{d}$=0.3 1/d, $z$=0.1 TU and $b$=9.0 1/TU/d. We can transform all the parameters back into the original mol/L unit by using the TU conversion factor whatever TU is present in the unit.

The dominant rate constant $k_{d}$=0.3 1/d is independent of TU, thus no conversion is required.

Threshold $z$=0.1 TU is dependent on TU and can be transformed back using the TU conversion factor resulting in 0.1 TU * 200 mol/L/TU = 20 mol/L

Killing rate $b$=9.0 1/TU/d is also dependent on TU and is transformed back by calculating:

$\frac{9.0 1/TU/d}{200 mol/L/TU}=0.045 L/mol/d$

Different models and different exposures can have very different exposure units. SI Table S2 shows some example units for concentrations, body burdens and food units and their toxic unit counterpart.

# Example units for model parameters assuming different exposure units

SI Table S2 Example units for GUTS-RED and BufferGUTS parameters assuming different exposure units. Parameters and state variables in toxic units (TU) can be converted back to their original unit using the LD50 or LC50.

| Parameters and state variables | Concentration unit | Body burden unit | Food unit | Toxic unit |
| --- | --- | --- | --- | --- |
| Reduced GUTS | | | | |
| Concentration $C$ | mol/L  g/L | g/Individual  mol/kg  g/kg  [compound/body mass] | mg/kg  [compound/overall food] | TU |
| Dominant rate constant $k_{d}$ | 1/d | 1/d | 1/d | 1/d |
| Damage $D$ | mol/L  g/L | g/Individual  mol/kg  g/kg  [compound/body mass] | mg/kg  [compound/overall food] | TU |
| Threshold $z$ or median threshold $\alpha$ | mol/L  g/L | g/Individual  mol/kg  g/kg  [compound/body mass] | mg/kg  [compound/overall food] | TU |
| Killing rate $b$ | L/mol/d  L/g/d | Individual/g/d  Kg/mol/d  Kg/g/d  [body mass/ compound/ day] | Kg/mg/d  [overall food/ compound/ day] | 1/TU/d |
| Shape parameter $\beta$ | [-] | [-] | [-] | [-] |
| Background mortality $h_{b}$ | 1/d | 1/d | 1/d | 1/d |
| Survival $S$ | [-] | [-] | [-] | [-] |
| Buffer GUTS specific | | | | |
| Buffer speed $\eta$ | 1/d | 1/d | 1/d | 1/d |
| Buffer $B$ | mol/L  g/L | g/Individual  mol/kg  g/kg  [compound/body mass] | mg/kg  [compound/overall food] | TU |

# Additional figures and tables

## Model quality metrics


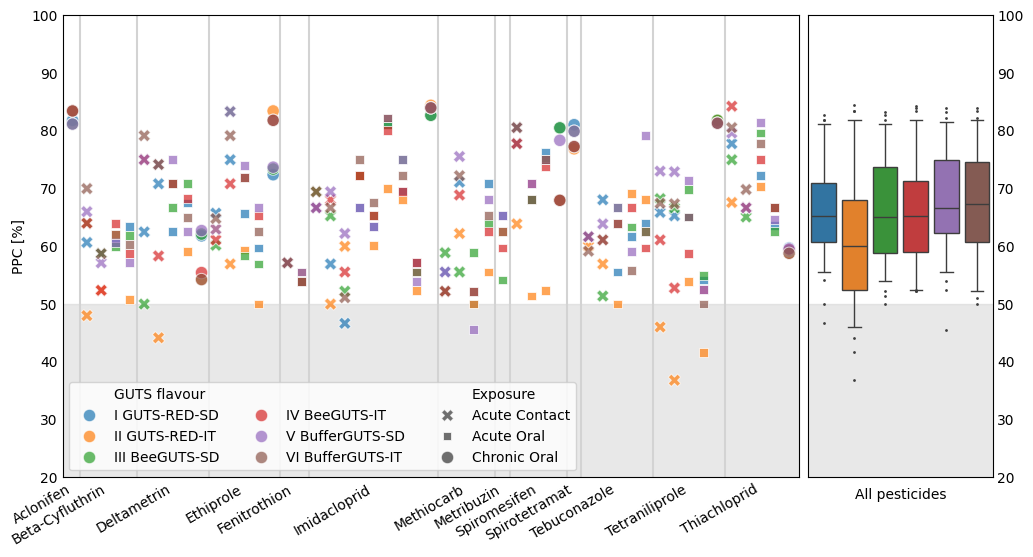


SI Figure S4: Posterior predictive check (PPC) model performance metric over all 51 datasets as described in EFSA SO (EFSA PPR, 2018). Dark-grey areas indicate a poor model performance according to the EFSA SO and light-grey according to the BeeGUTS paper (Baas et al., 2022). Whiskers show 5 to 95 percentiles.


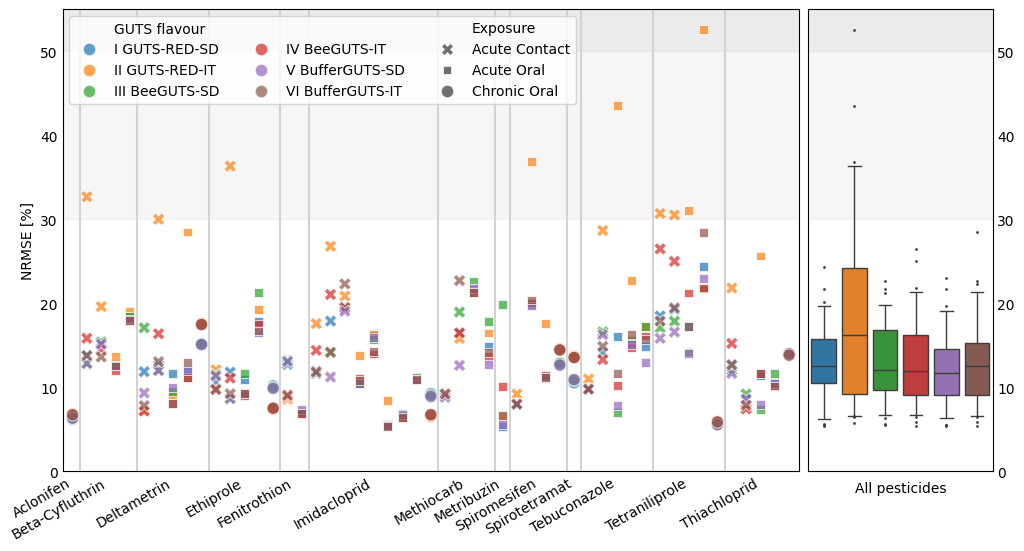


SI Figure S5: Normalized root mean square error (NRMSE) model performance metric over all 51 datasets as described in EFSA SO (EFSA PPR, 2018). Dark-grey areas indicate a poor model performance according to the EFSA SO and light-grey according to the BeeGUTS paper (Baas et al., 2022). Whiskers show 5 to 95 percentiles.


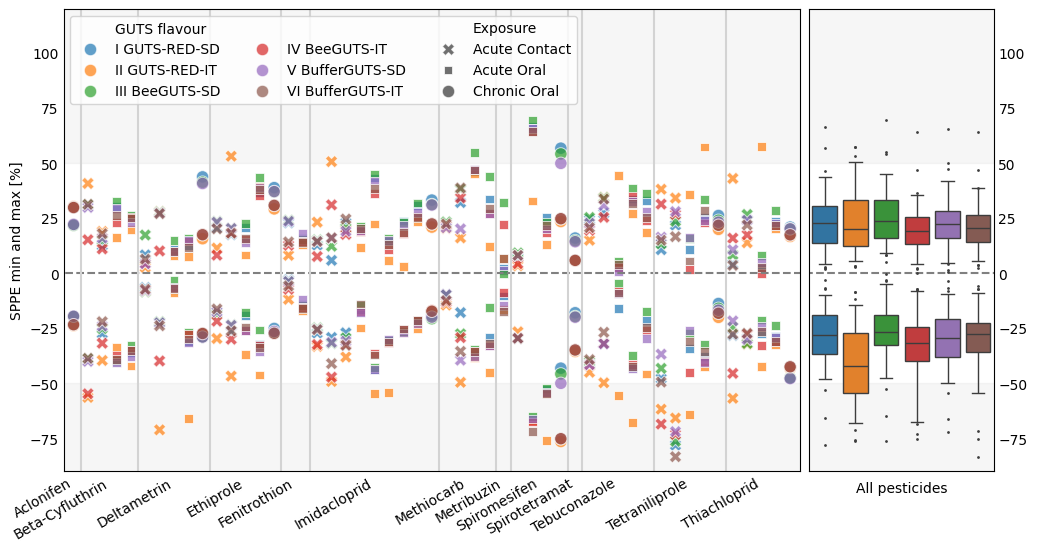


SI Figure S6: Survival probability prediction error (SPPE) minimum(bottom) and maximum (top) model performance metric over all 51 datasets as described in EFSA SO (EFSA PPR, 2018). Dark-grey areas indicate a poor model performance according to the EFSA SO and light-grey according to the BeeGUTS paper (Baas et al., 2022). Whiskers show 5 to 95 percentiles.

## Parameter values plotted per exposure route


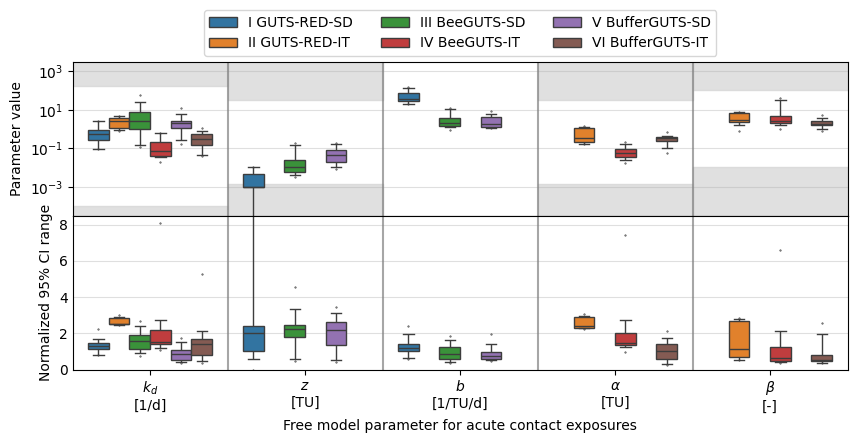


SI Figure S7: Calibrated parameter values (top) and 95% credibility interval (CI) ranges normalized by the parameter value (bottom) for all tested models and only the 19 datasets with acute contact exposures. Some parameters are given in toxic units (TU) and background mortality hb values are not shown. Grey areas are unlikely parameter ranges based on Delignette-Muller et al. (2017) derived for all datasets. Whiskers show 5 to 95 percentiles, the whisker for the threshold z of the GUTS-RED-SD model goes down to the minimum parameter value of 10-10.


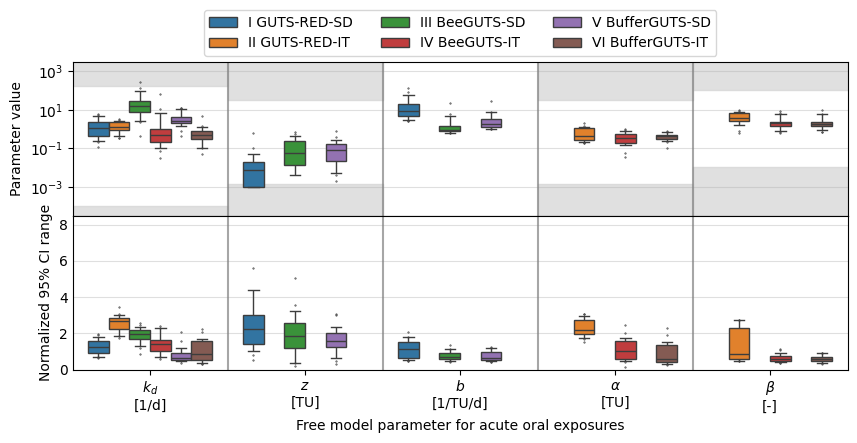


SI Figure S8: Calibrated parameter values (top) and 95% credibility interval (CI) ranges normalized by the parameter value (bottom) for all tested models and only the 24 datasets with acute oral exposures. Some parameters are given in toxic units (TU) and background mortality hb values are not shown. Grey areas are unlikely parameter ranges based on Delignette-Muller et al. (2017) derived for all datasets. Whiskers show 5 to 95 percentiles.


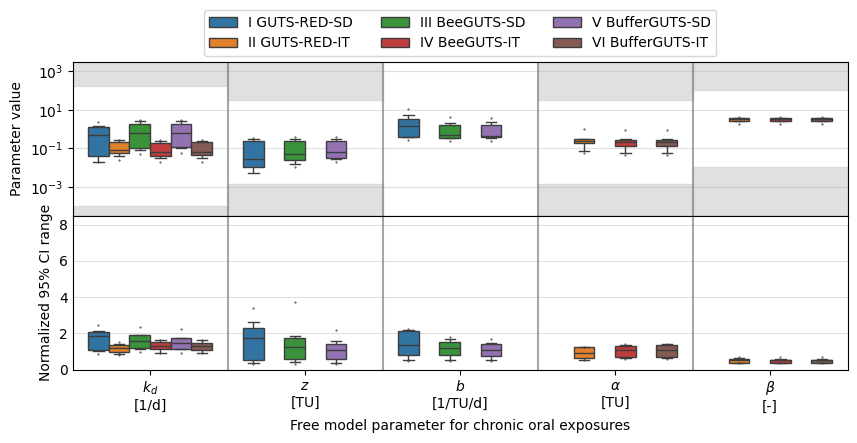


SI Figure S9: Calibrated parameter values (top) and 95% credibility interval (CI) ranges normalized by the parameter value (bottom) for all tested models and only the eight datasets with chronic oral exposures. Some parameters are given in toxic units (TU) and background mortality hb values are not shown. Grey areas are unlikely parameter ranges based on Delignette-Muller et al. (2017) derived for all datasets. Whiskers show 5 to 95 percentiles.

## Model calibration benchmark results for GUTS ring-test data

Our Bayesian calibration procedure is implemented in Python and not optimized for speed. Thus, calibrations were performed on the High Performance Cluster (HPC) of the Osnabrück University, Germany.

SI Table S3 Comparison to the GUTS implementation OpenGUTS (openguts.info) and MORSE (Baudrot & Charles, 2021, mosaic.univ-lyon1.fr/guts) using the ringtest datasets A and B (dataset C was not used because our version cannot yet work with datasets with timepoints without observations). Shown values are the optimal value with the 95% confidence or credibility intervals (CI) in parenthesis. Datasets A SD and IT are artificial datasets, therefore results can be compared to the original value used to generate the datasets. The pulsed experiments were not reliably calibratable, as indicated by the asterisk in the OpenGUTS fit (identifying boundary problems in the CI calculation) and many divergences (indicating MCMC chains to be stuck in regions which cannot be adequately explored) in our Bayesian fit.

| **Dataset (Model)** | **Parameter** | **Our fit** | **OpenGUTS** | **MORSE** | **Original** |
| --- | --- | --- | --- | --- | --- |
| Data A SD (SD) | $k_{d}$ | 0.691 (0.457-0.946) | 0.712 (0.497-0.981) | 0.702 (0.495-0.975) | 0.8 |
|  | $z$ | 2.80 (2.22-3.31) | 2.89 (2.29-3.36) | 2.89 (2.29-3.31) | 3 |
|  | $b$ | 0.640 (0.371-0.951) | 0.619 (0.414-1.09) | 0.619 (0.404-0.983) | 0.6 |
|  | $h_{b}$ | 0.007 (0.0-0.017) | 0.008 (0.00138-0.0253) | 0.0077 (0.0015-0.0228) | 0.01 |
| Data A IT (IT) | $k_{d}$ | 0.786 (0.514-1.06) | 0.793 (0.558-1.11) | 0.769 (0.535-1.08) | 0.8 |
|  | $\alpha$ | 5.36 (4.38-6.36) | 5.42 (4.48-6.41) | 5.35 (4.4-6.35) | 5 |
|  | $\beta$ | 5.08 (3.38-6.91) | 5.19 (3.71-7.38) | 5.0 (3.52-7.03) | 5 |
|  | $h_{b}$ | 0.025 (0.007-0.046) | 0.0262 (0.0102-0.0518) | 0.0242 (0.00929-0.0485) | 0.02 |
| Data B Const (SD) | $k_{d}$ | 2.29 (1.49-3.30) | 2.16 (1.60-3.33) | 2.19 (1.58-3.49) | - |
|  | $z$ | 16.9 (15.4-18.6) | 17.1 (15.9-17.7) | 16.9 (15.4-18.6) | - |
|  | $b$ | 0.127 (0.075-0.185) | 0.132 (0.0863-0.196) | 0.123 (0.0781-0.189) | - |
|  | $h_{b}$ | 0.027 (0.011-0.046) | 0.0276 (0.0133-0.0495) | 0.0268 (0.0127 – 0.0489) | - |
| Data B Const (IT) | $k_{d}$ | 0.723 (0.52-0.939) | 0.750 (0.555-0.977) | 0.719 (0.528-0.941) | - |
|  | $\alpha$ | 17.7 (15.0-20.3) | 18.1 (15.4-20.6) | 17.7 (15.1-20.2) | - |
|  | $\beta$ | 6.68 (4.69-8.86) | 7.04 (5.20-9.41) | 6.69 (4.91-9.06) | - |
|  | $h_{b}$ | 0.016 (0.0-0.032) | 0.0186 (0.00491-0.0415) | 0.0158 (0.00374-0.0375) | - |
| Data B Puls (SD) | $k_{d}$ | 4.481 (1.524-8.624) | 2.23 (1.37-144*) | *Did not produce results in 24h* | - |
|  | $z$ | 17.999 (0.0-24.796) | 22.5 (18.7-26.7) | *Did not produce results in 24h* | - |
|  | $b$ | 0.101 (0.003-0.271) | 0.458 (0.0465-1.46) | *Did not produce results in 24h* | - |
|  | $h_{b}$ | 0.023 (0.016-0.03) | 0.024 (0.0175-0.0319) | *Did not produce results in 24h* | - |
| Data B Puls (IT) | $k_{d}$ | 16.027 (0.0-90.096) | 0.924 (0.516-144*) | 0.452 (0.000685-18.1) | - |
|  | $\alpha$ | 35.255 (0.001-71.982) | 17.0 (16.3-34.2) | 17.6 (0.27-38.6) | - |
|  | $\beta$ | 35.255 (0.001-71.982) | 21.6 (2.25-50.7) | 2.29 (0.482-23.8) | - |
|  | $h_{b}$ | 0.027 (0.019-0.034) | 0.0263 (0.0198-0.0341) | 0.0244 (0.0162-0.0331) | - |

## Calibration example for the non-Apis bee species Osmia bicornis


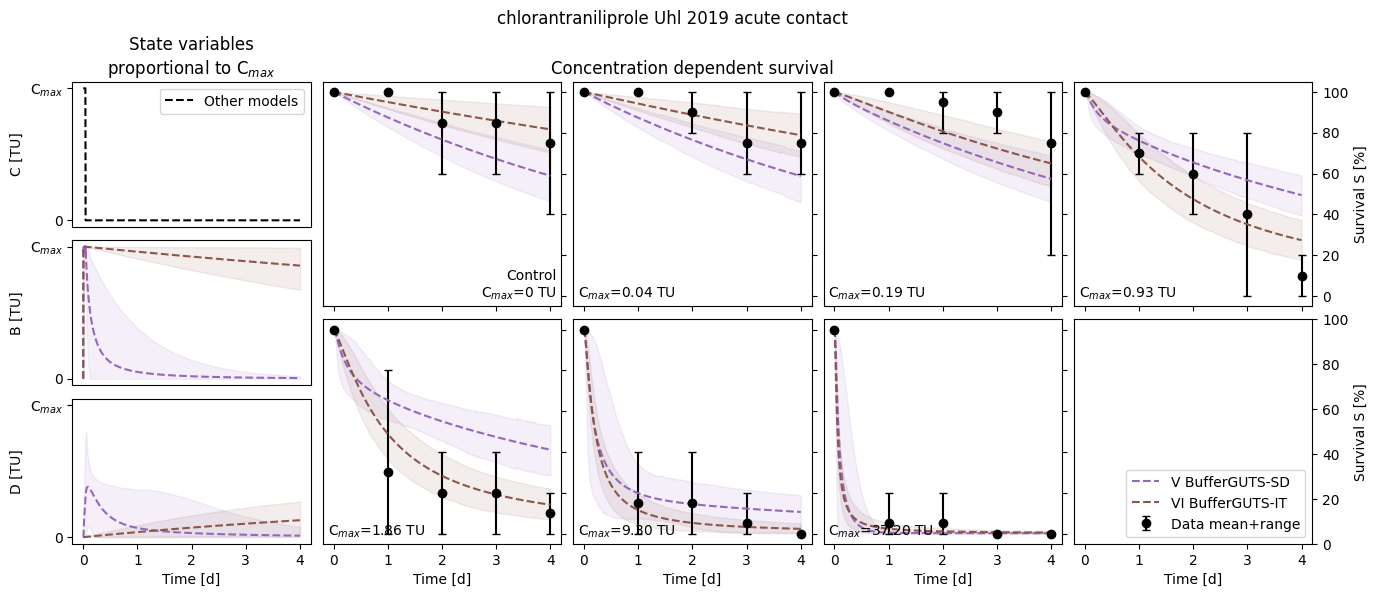


SI Figure S10: Exemplary calibration results for the non-Apis bee species Osmia bicornis for the substance Chlorantraniliprole from Uhl et al. (2019). Both models show a high background mortality $h_{b}$ due to high mortality in some of the control replicates and low mortality in the lower concentration treatments.

## Study Numbers of underlying toxicity data

Reports can be requested by sending an email to [cropscience-transparency@bayer.com](mailto:cropscience-transparency@bayer.com) or via their online form at <https://www.bayer.com/en/agriculture/safety-study-report-request-forms>.

SI Table S4 Substances and Study IDs of the regulatory studies from Bayer used to derive the datasets (CO=chronic oral, AC= acute contact, AO= acute oral).

| **Stubstance** | **Study ID / Document ID** | **Exposure** |
| --- | --- | --- |
| Aclonifen | M-601664  M-174936 | CO  AC & AO |
| Beta-cyfluthrin | M-051896  M-053813 | AC & AO  AC & AO |
| Deltamethrin | M-149494  M-149496  M-444971  M-477250 | AC  AO  AC & AO  CO |
| Ethiprole | M-192387  M-214951  M-581904 | AC & AO  AC & AO  CO |
| Fenitrothion | M-293568 | AC & AO |
| Imidacloprid | M-006940  M-016942  M-067751  M-067996  M-068023  M-600686 | AC & AO  AO  AC & AO  AO  AC & AO  CO |
| Methiocarb | M-357085  M-013166 | AC & AO  AC & AO |
| Metribuzin | M-014115  M-294086 | AC & AO  AC & AO |
| Spiromesifen | M-657628  M-031874  M-030406 | AC & AO  AC & AO  CO |
| Spirotetramat | M-298419  M-081227  M-395773  M-572046 | AC & AO  AC & AO  AC & AO  CO |
| Tebuconazole | M-105205  M-182469  M-103501 | AC & AO  AC & AO  AC & AO |
| Tetraniliprole | M-438810  M-441758  M-551955 | AC & AO  AC & AO  CO |
| Thiachloprid | M-000856  M-001004  M-475374 | AC & AO  AC & AO  CO |

## Complete calibration results


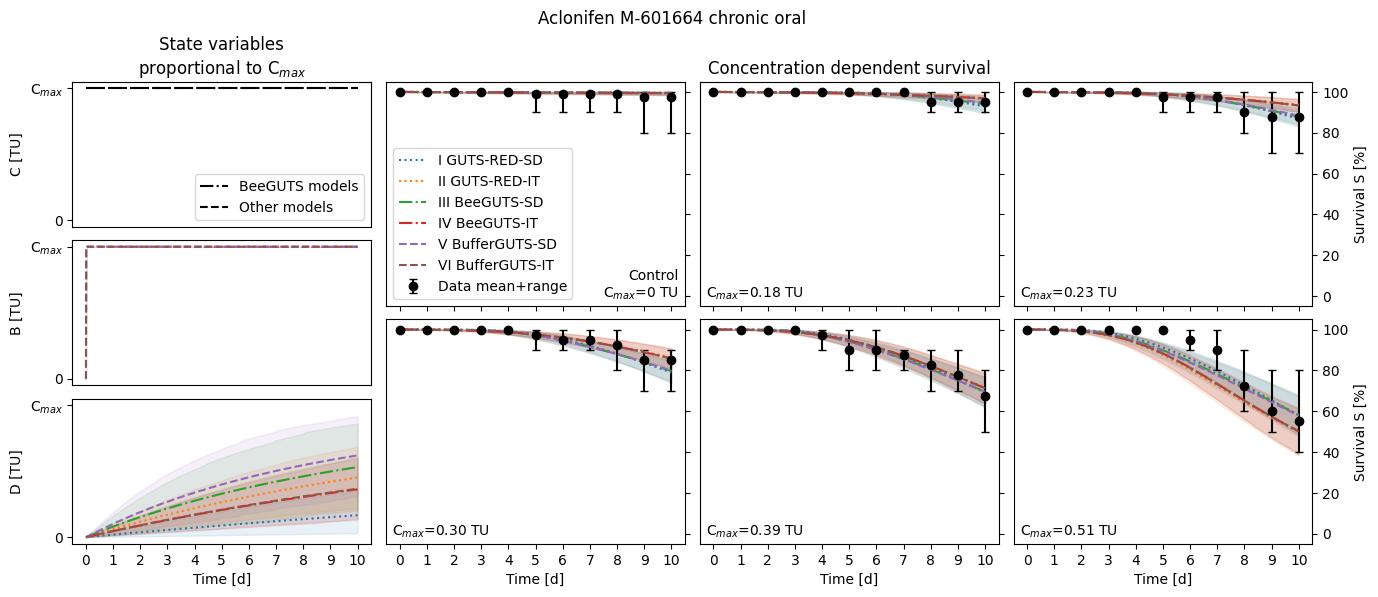


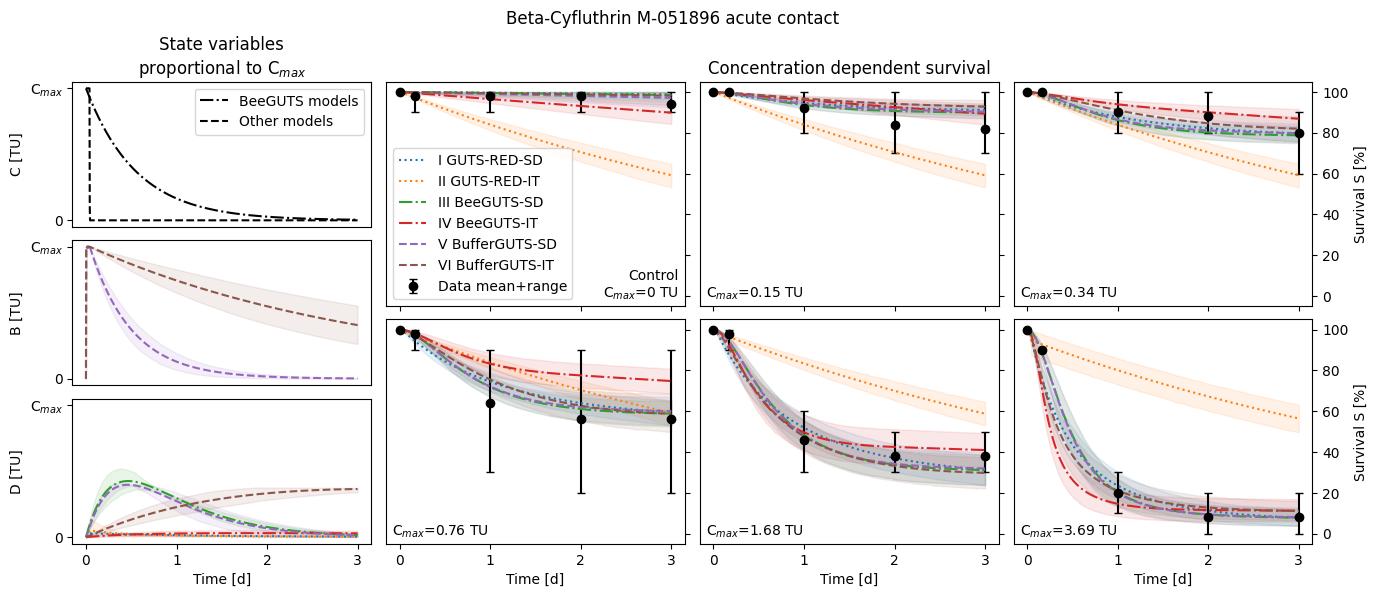


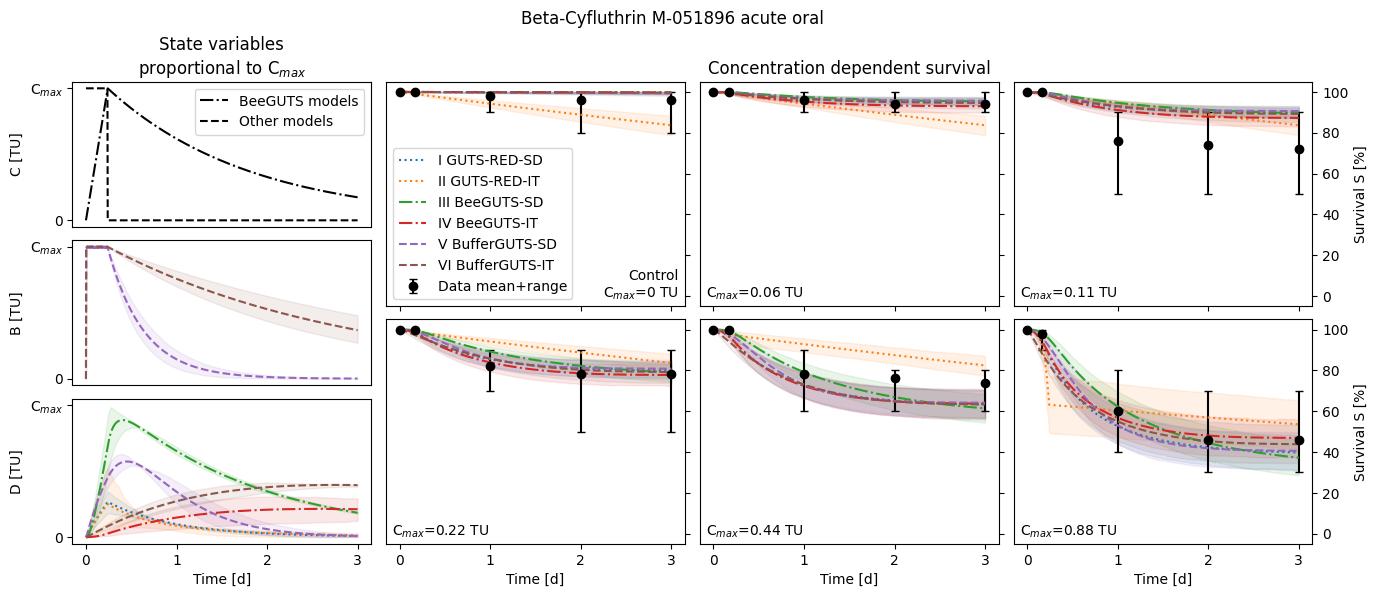


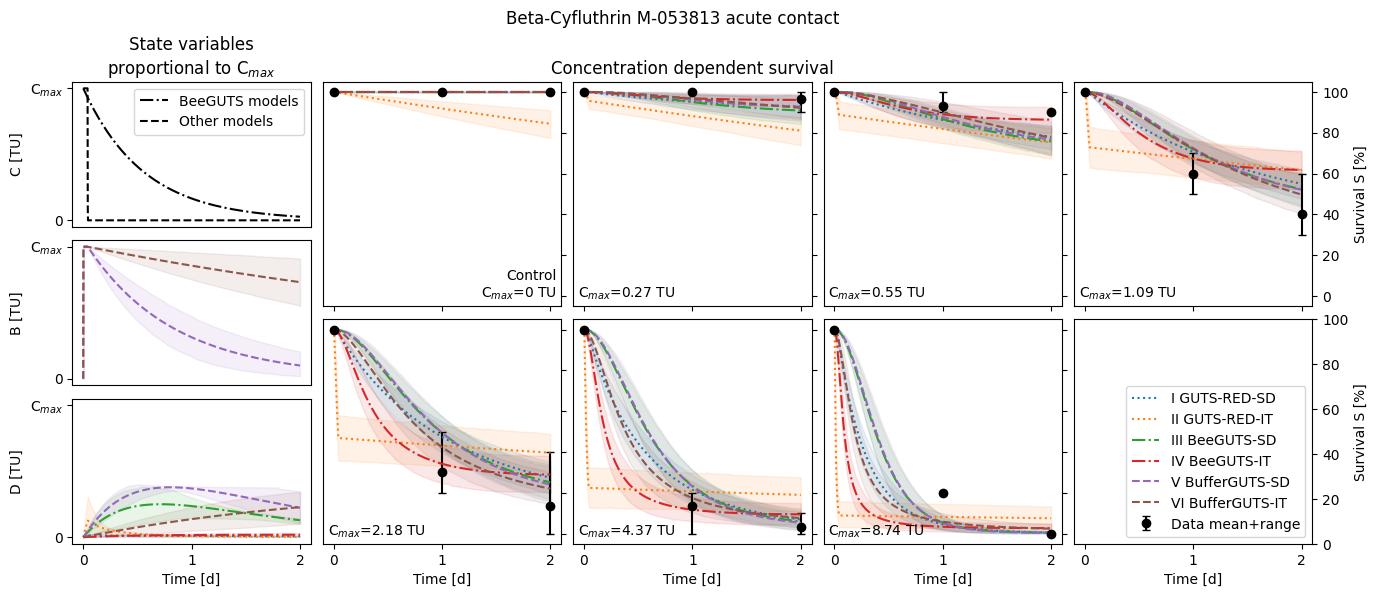


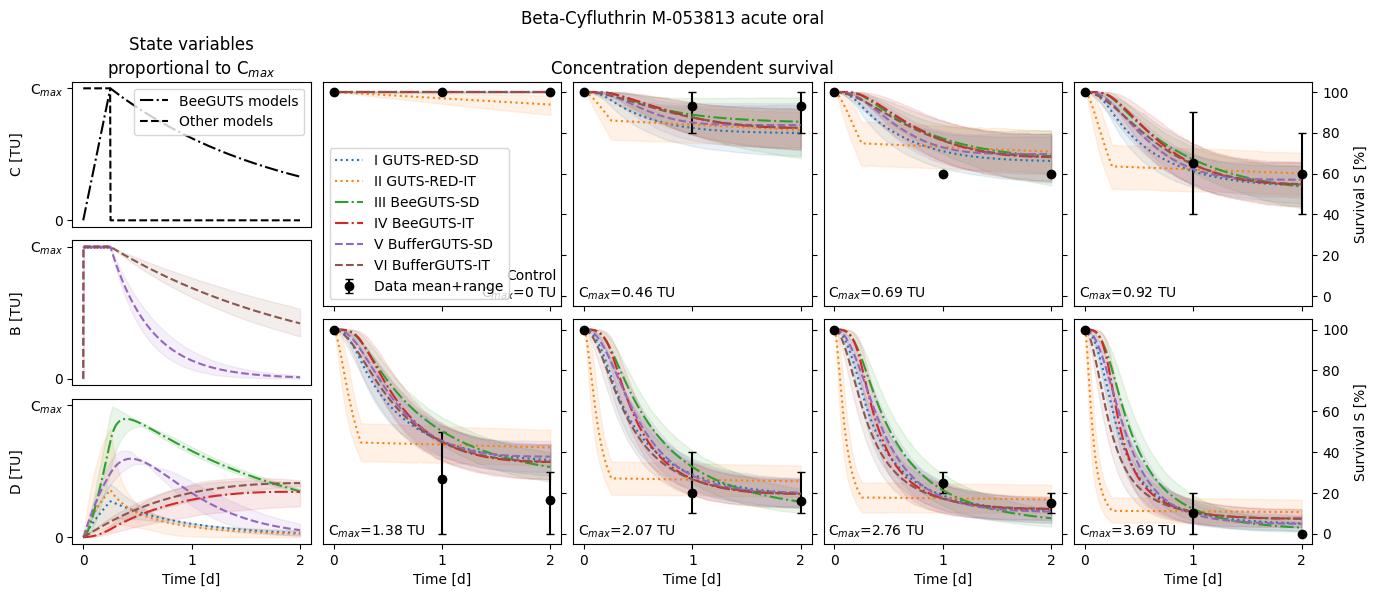


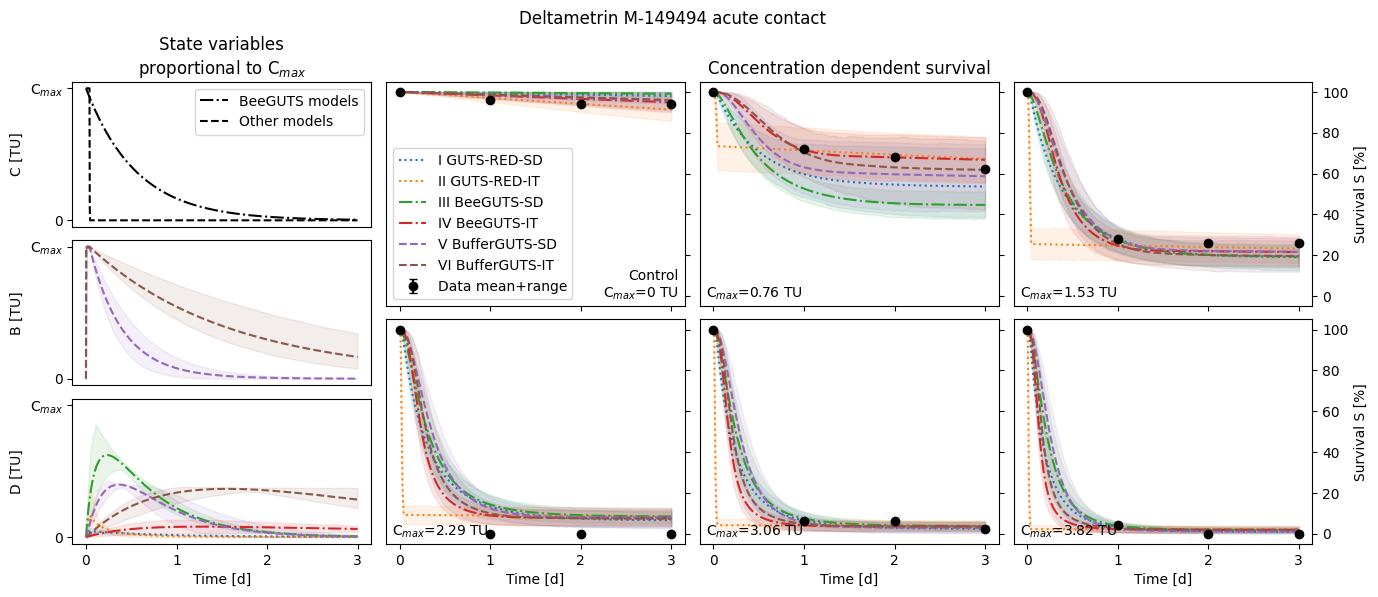


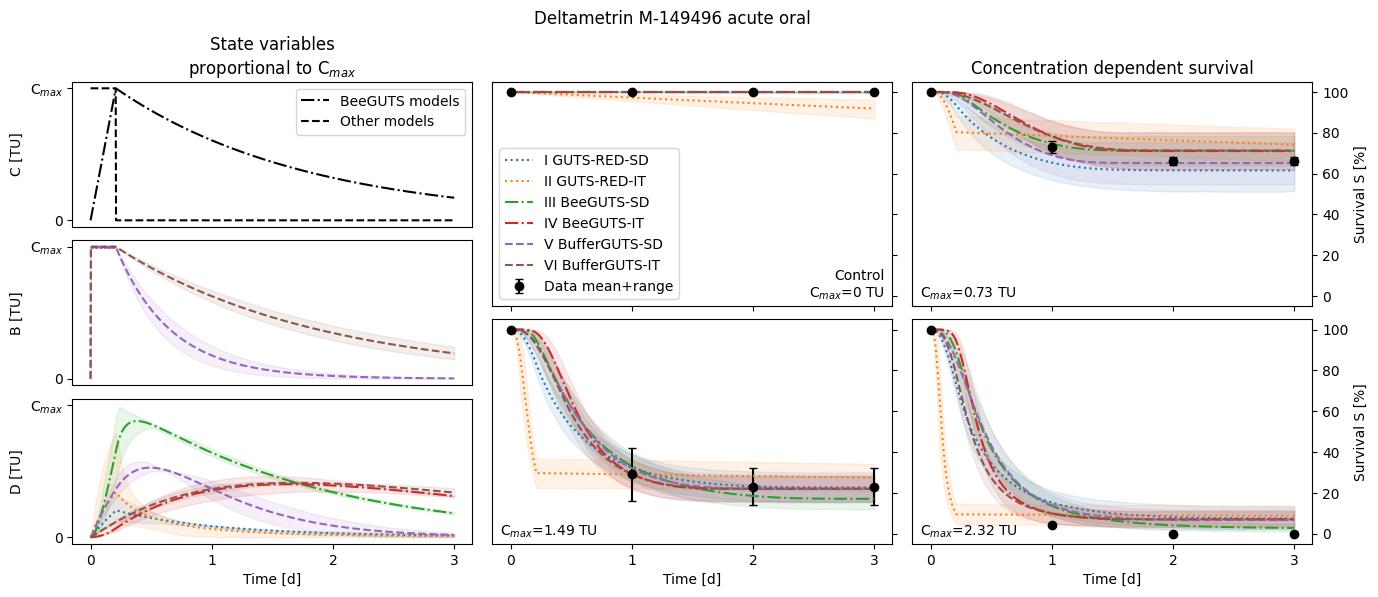

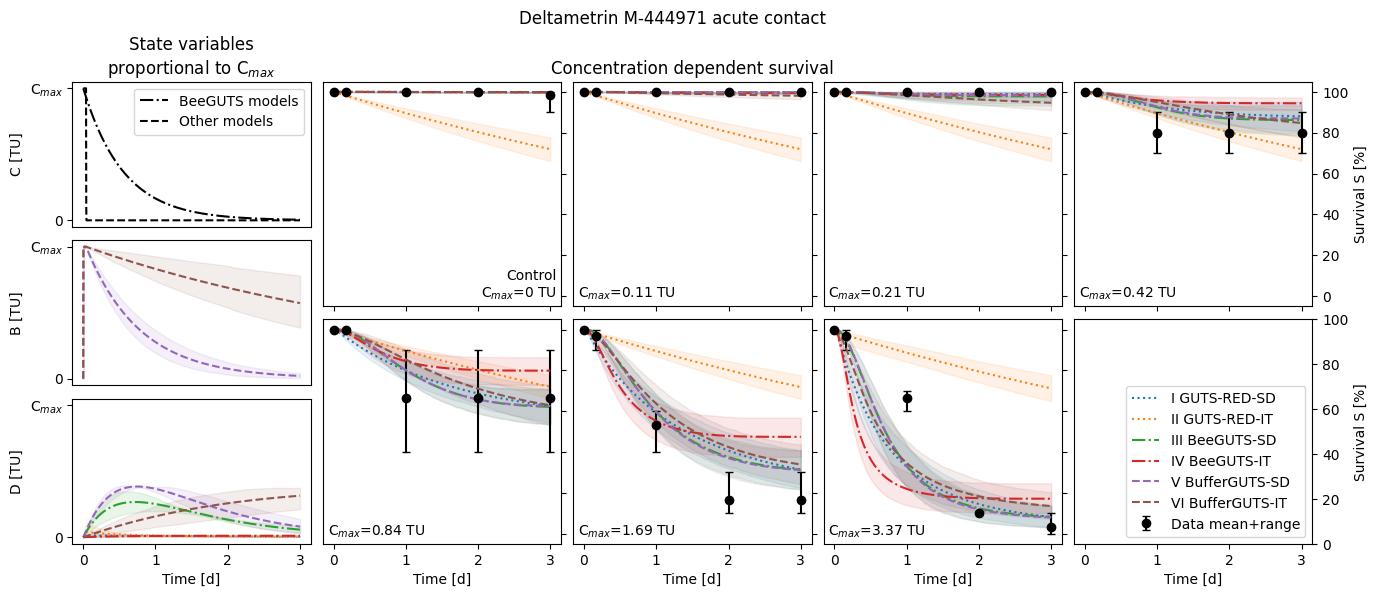

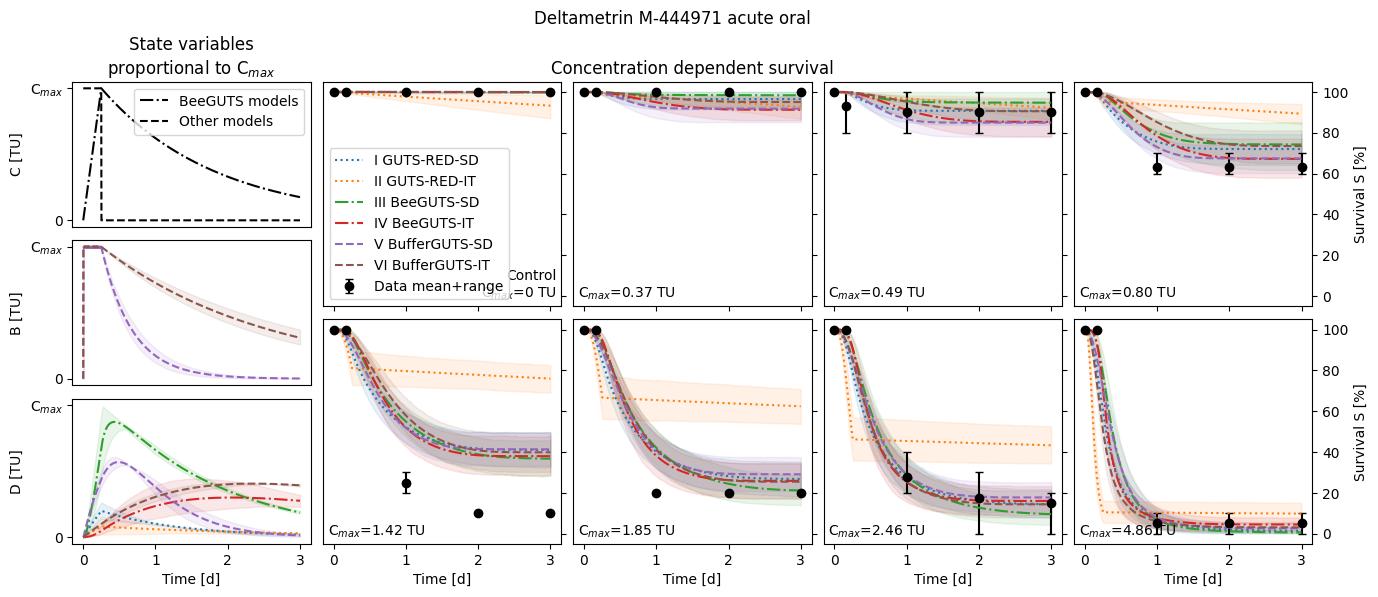

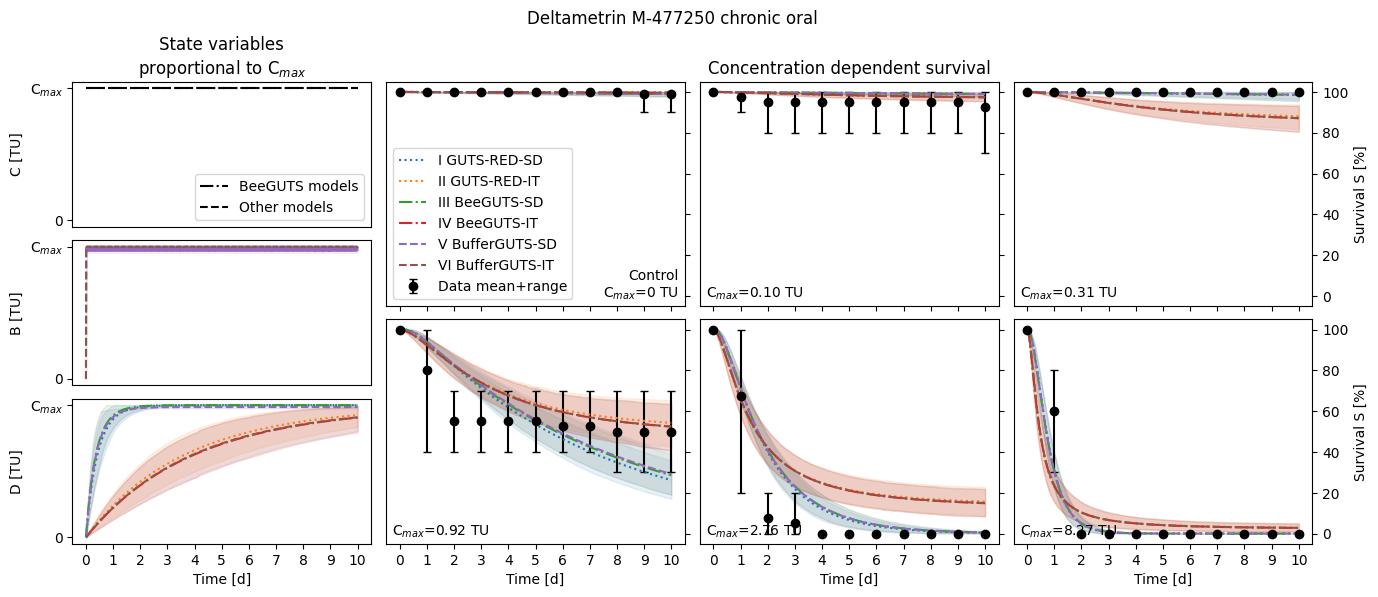

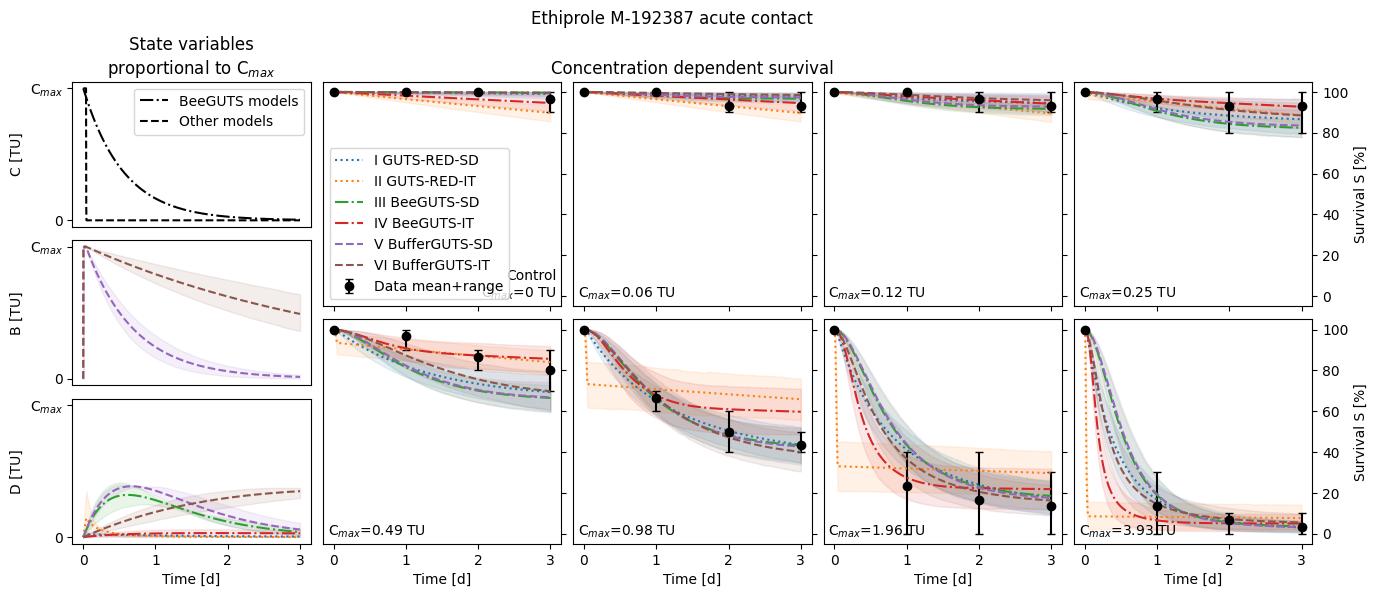

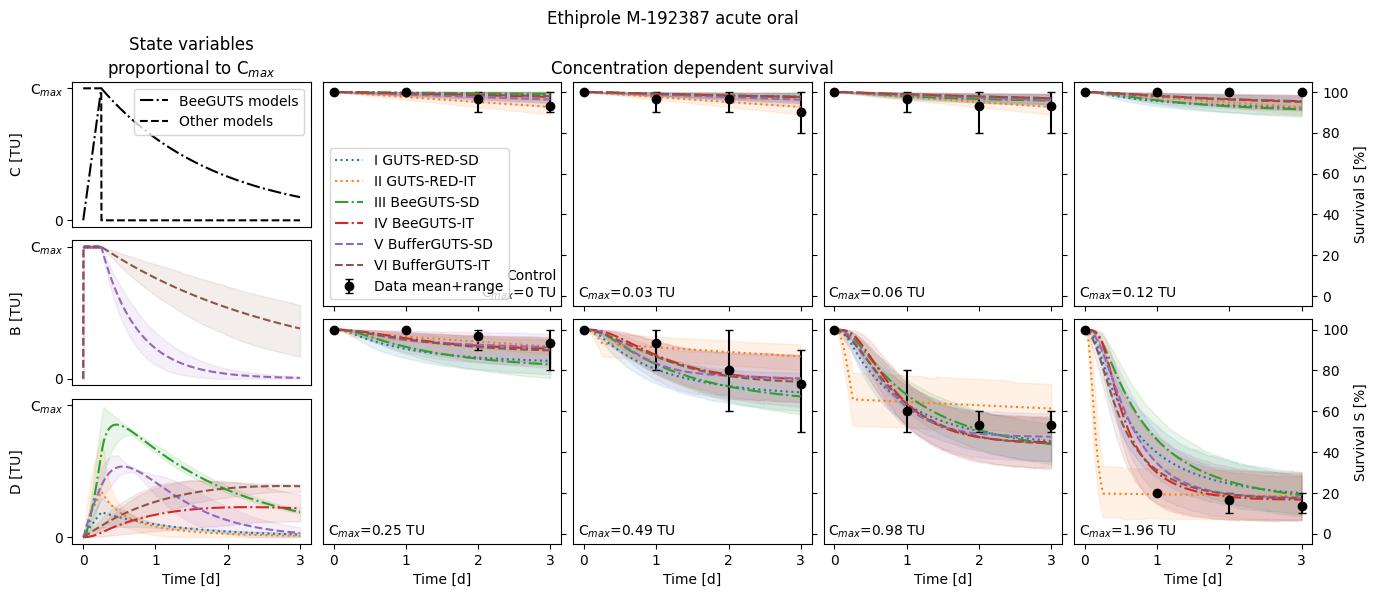

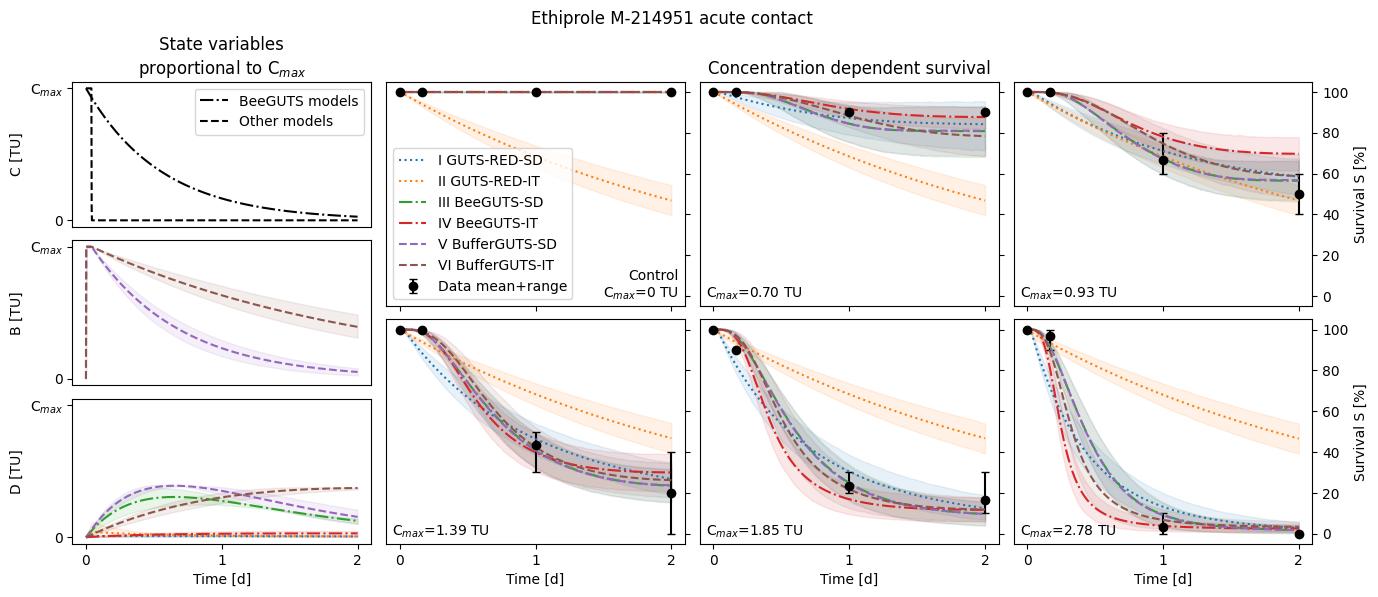

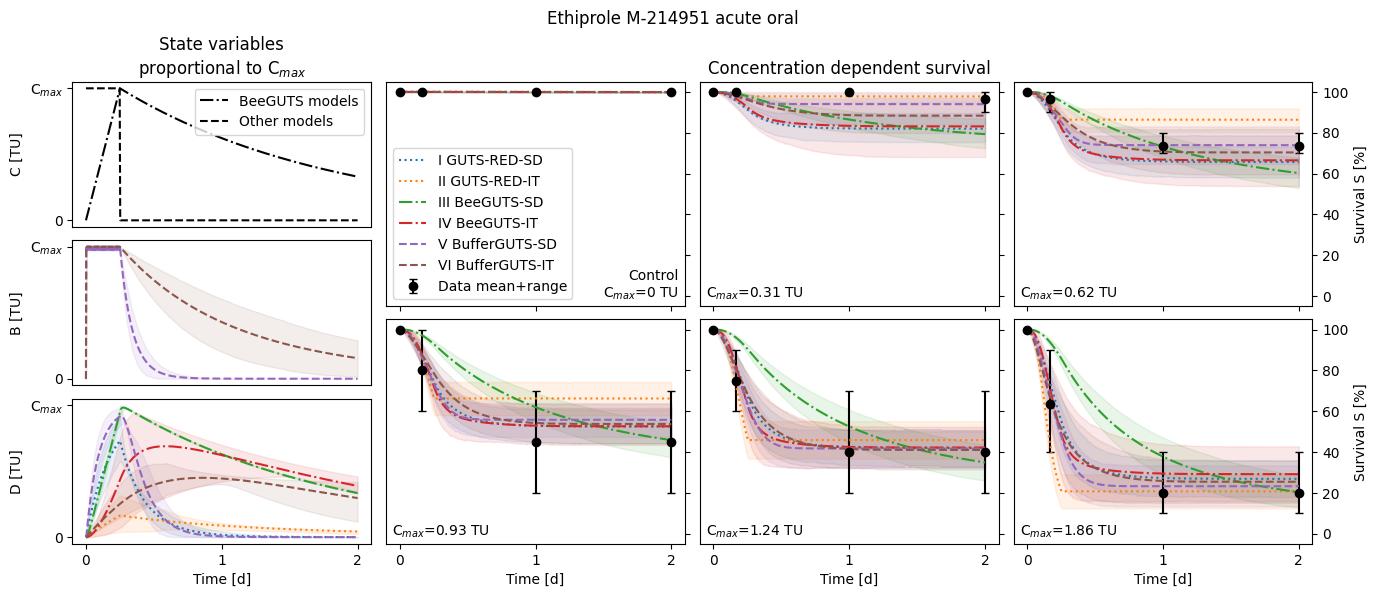

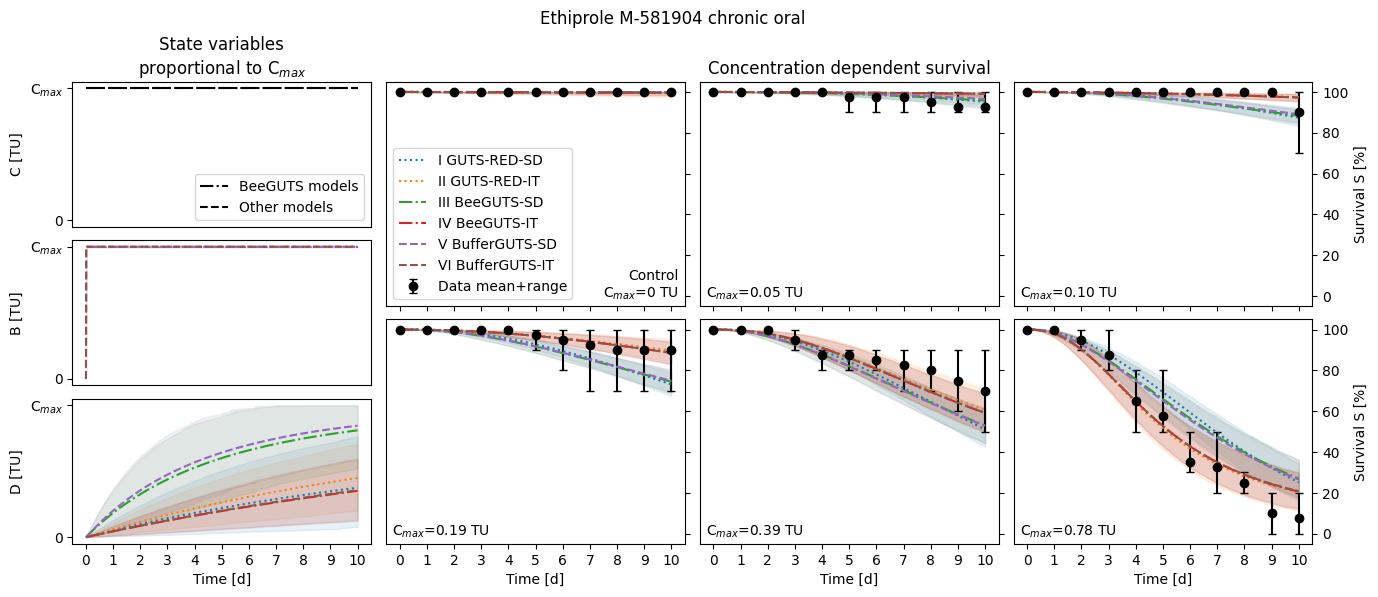

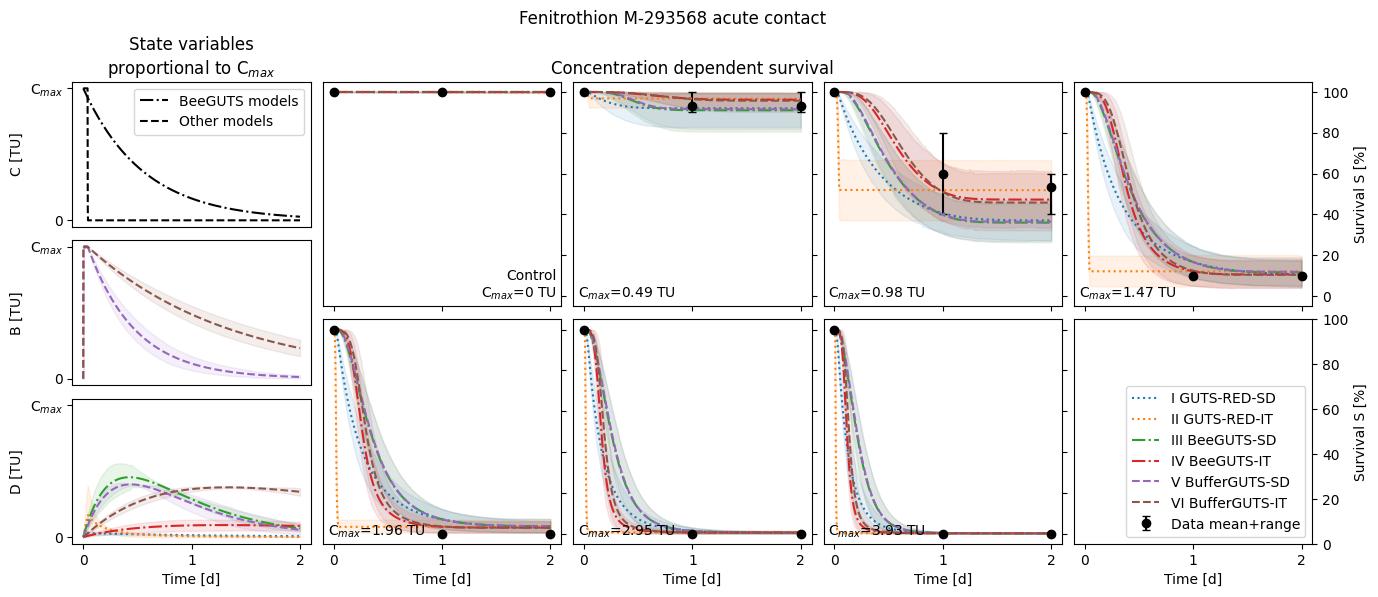

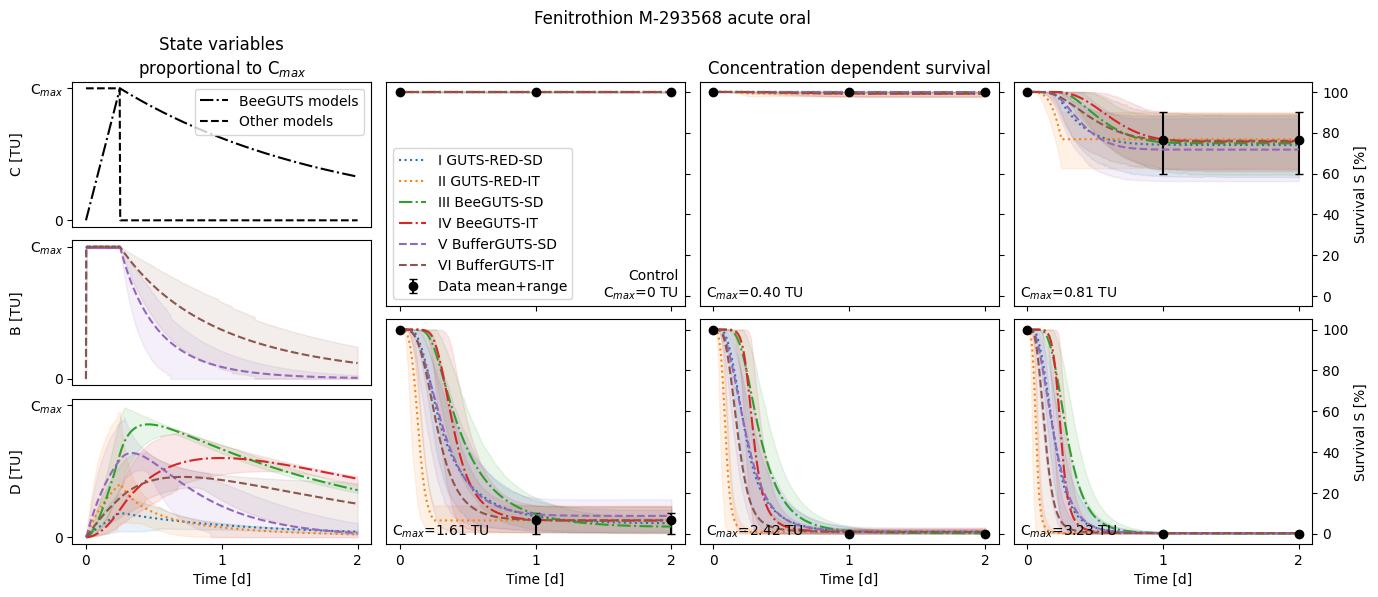

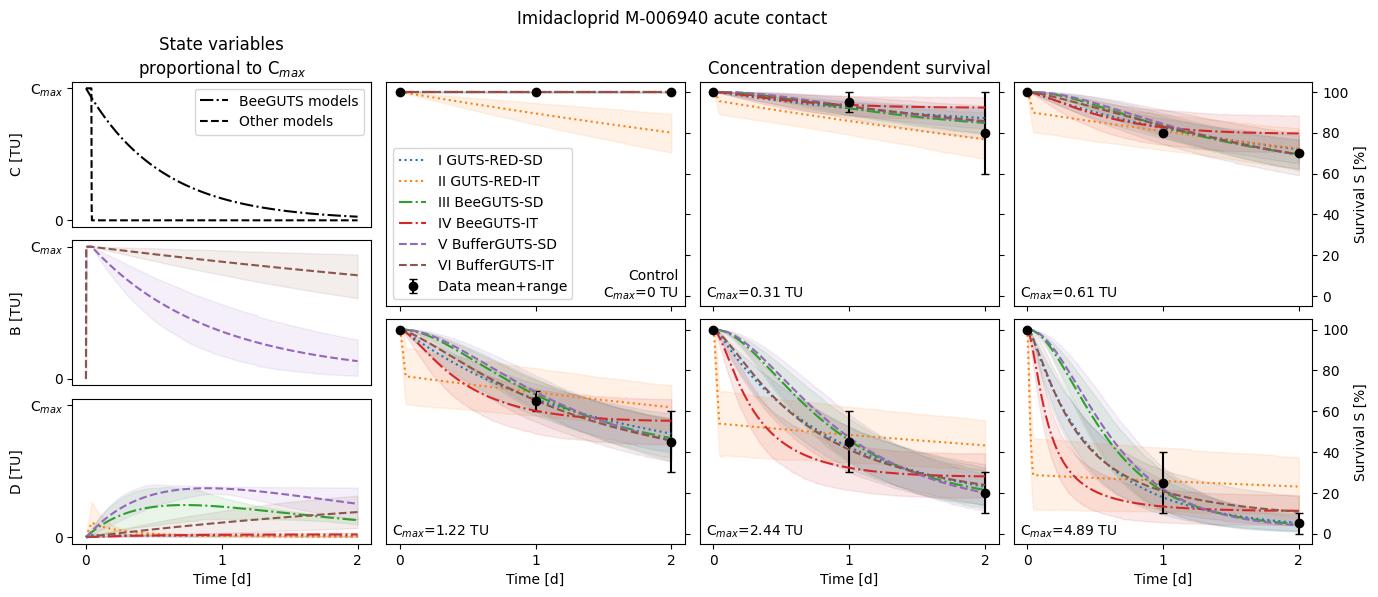

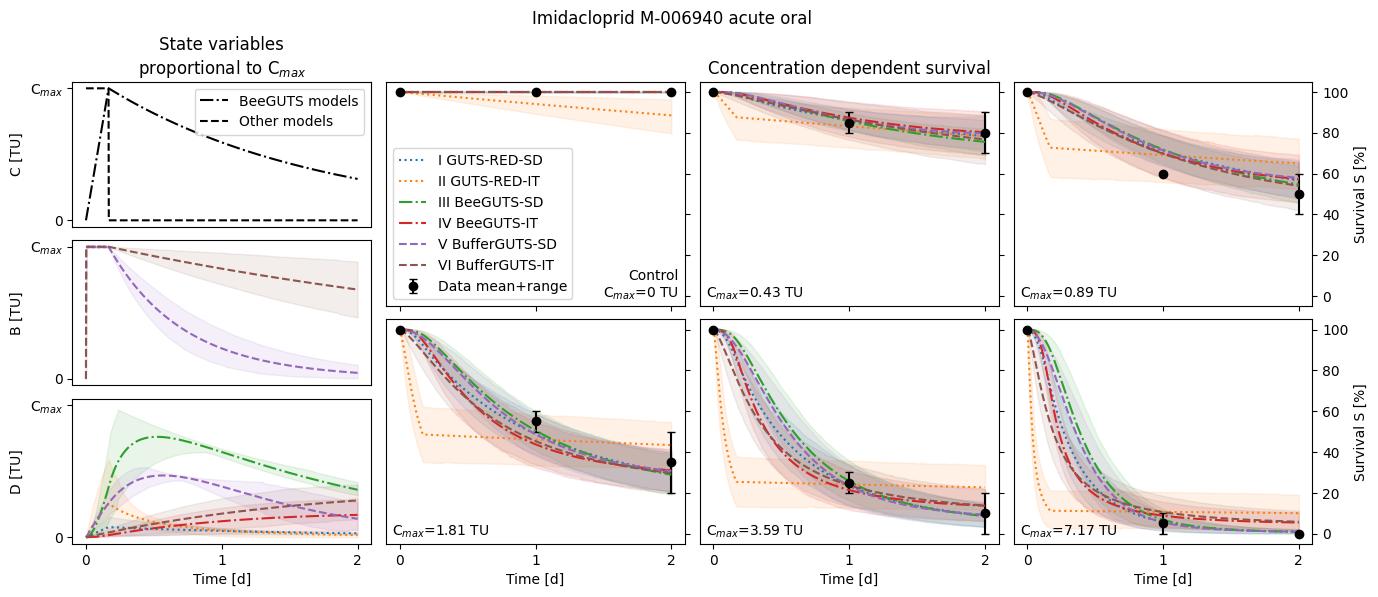

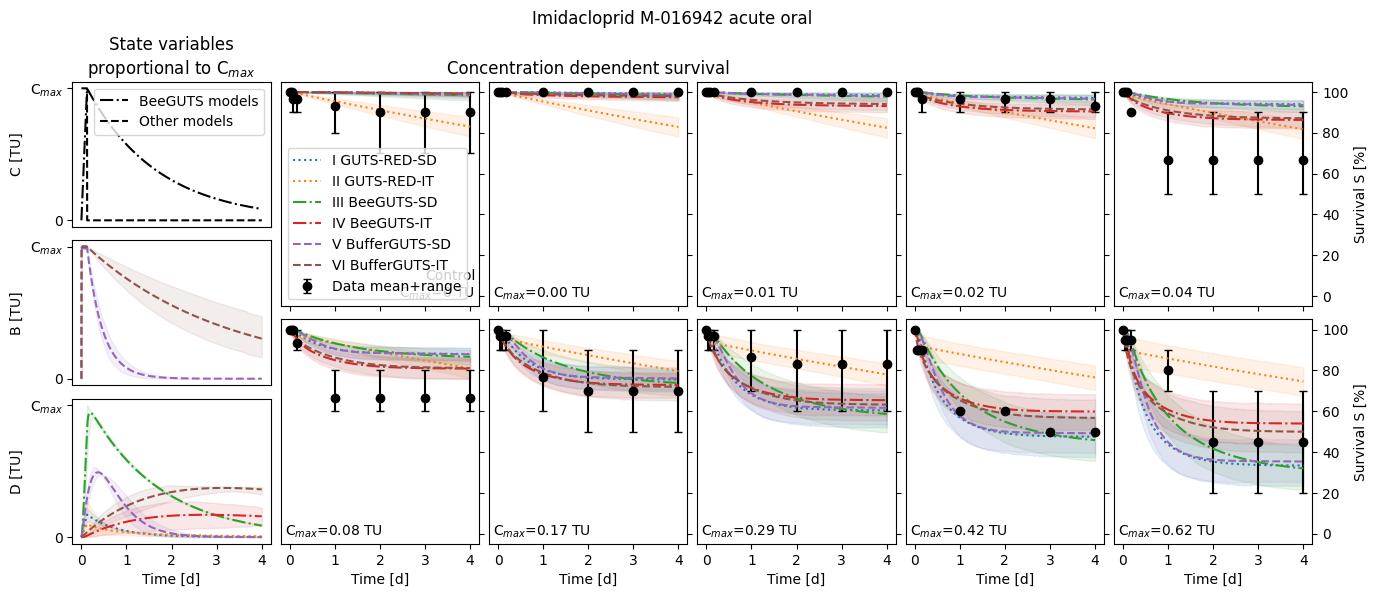

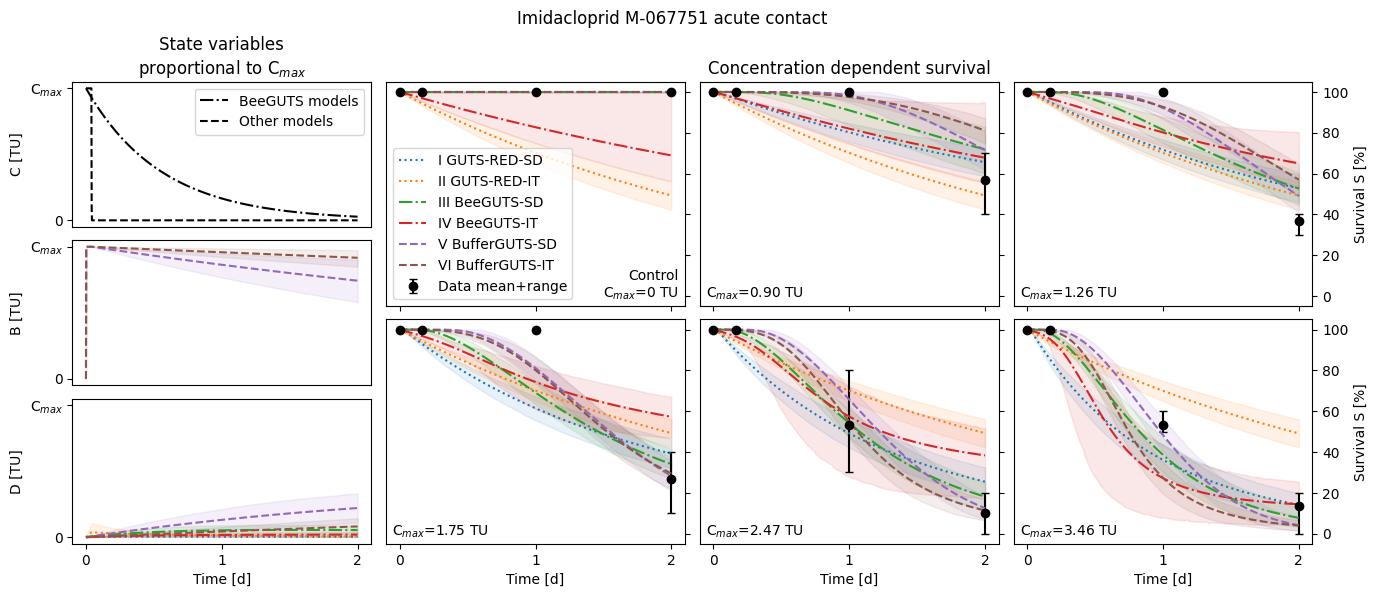

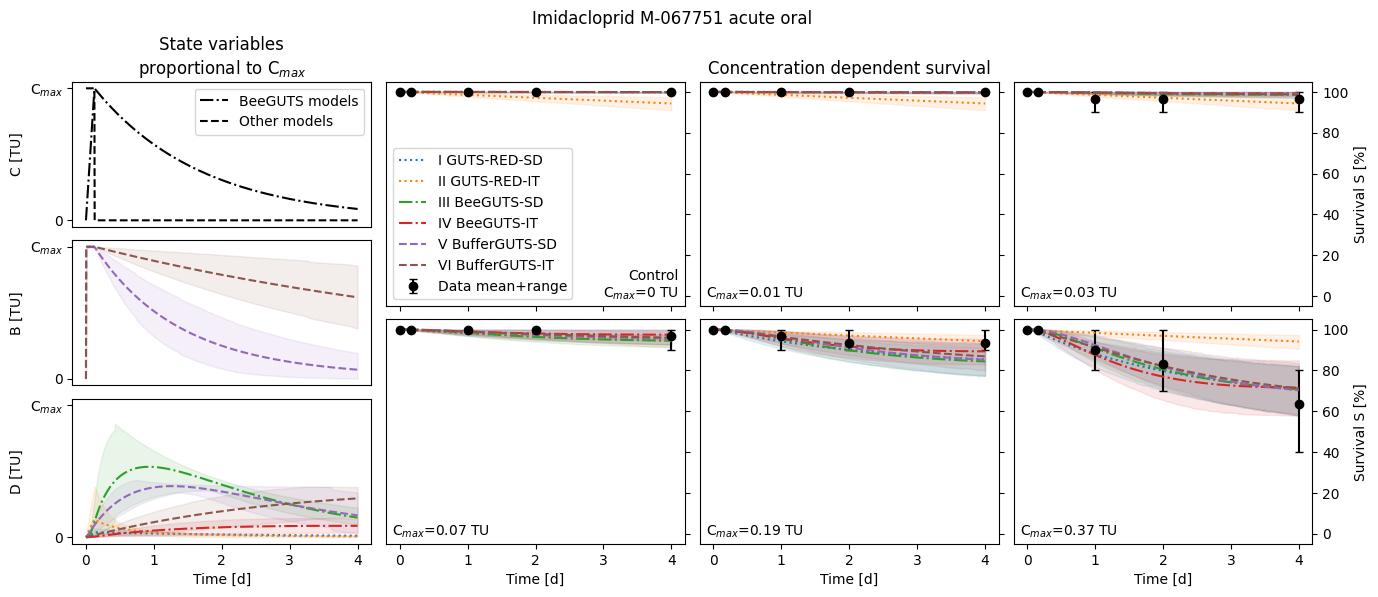

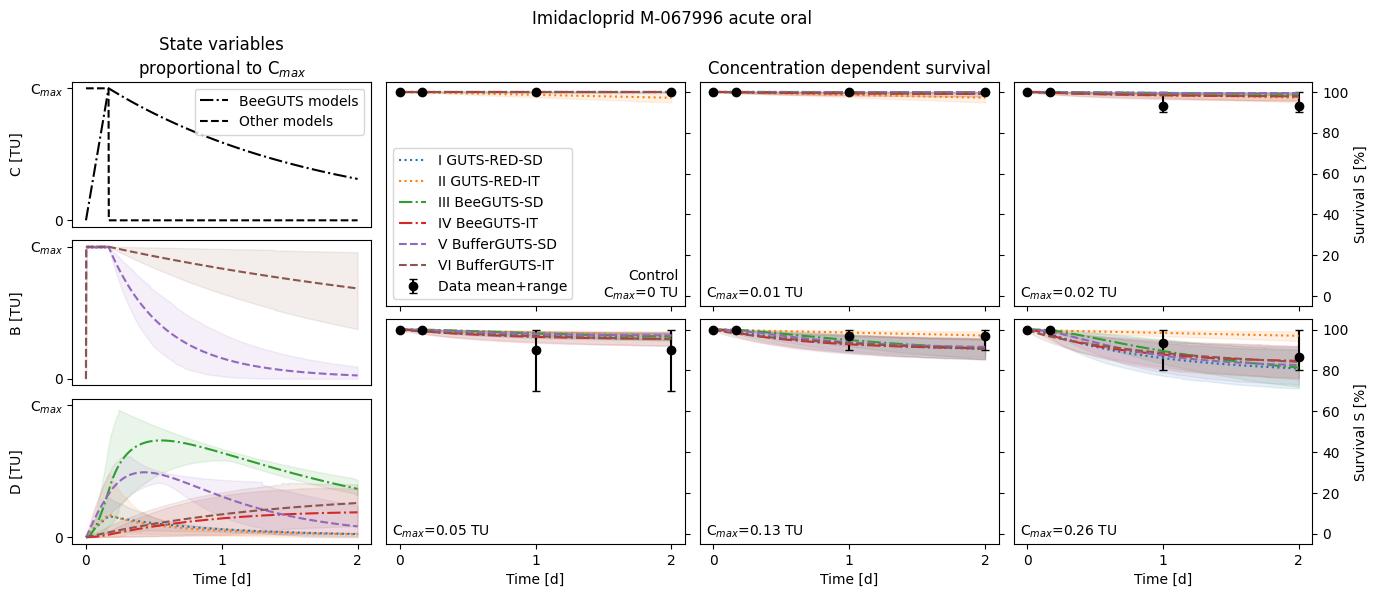

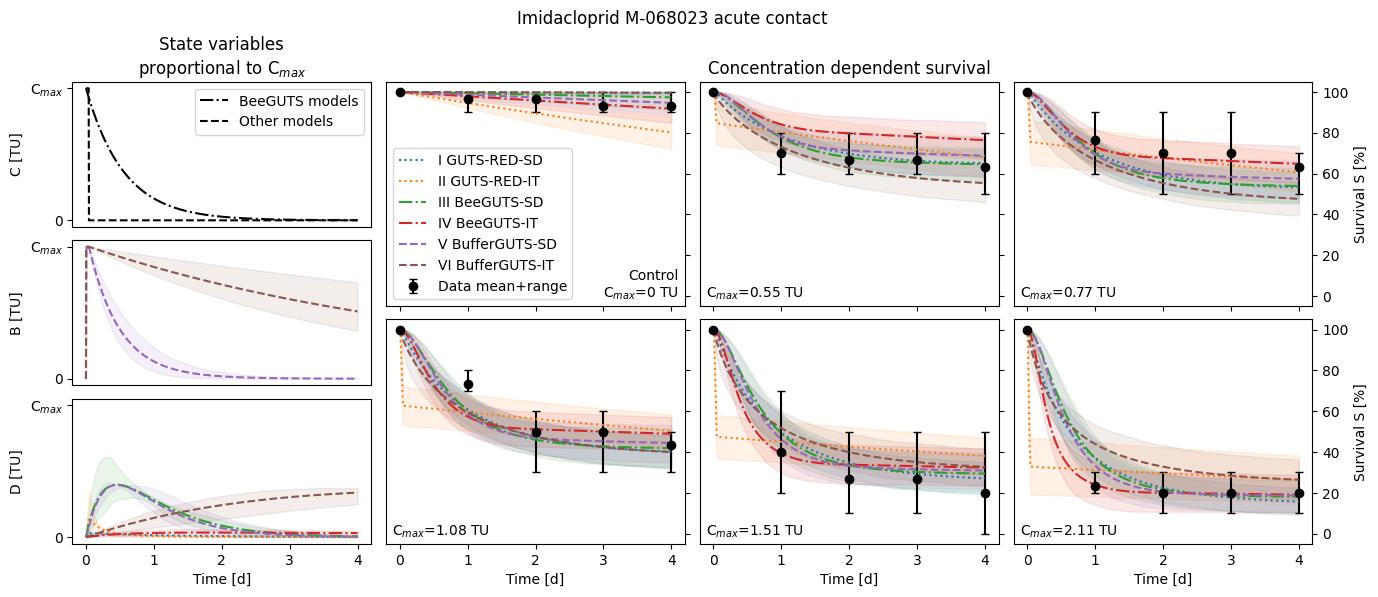

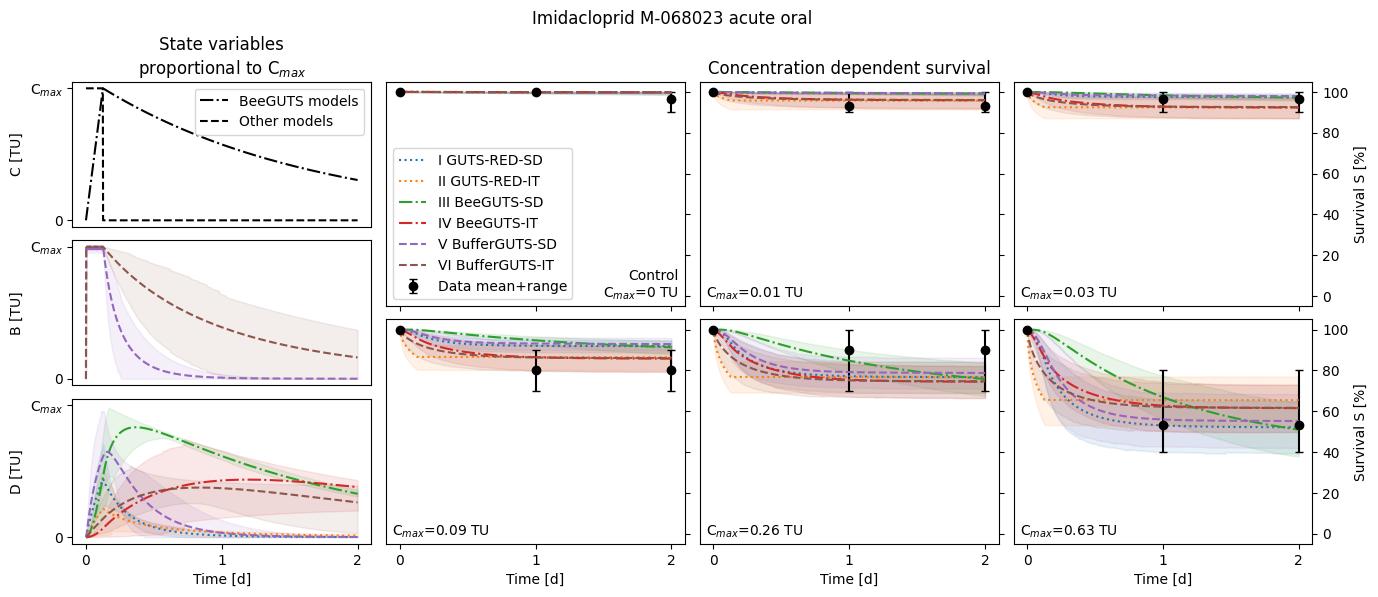

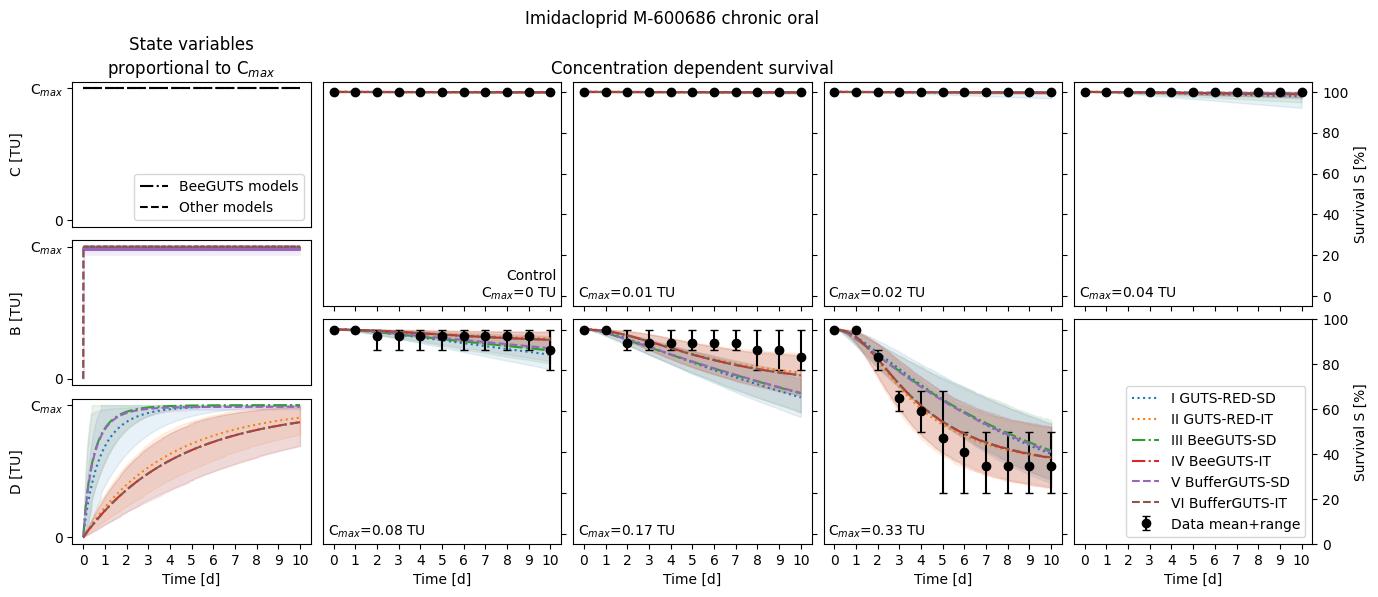

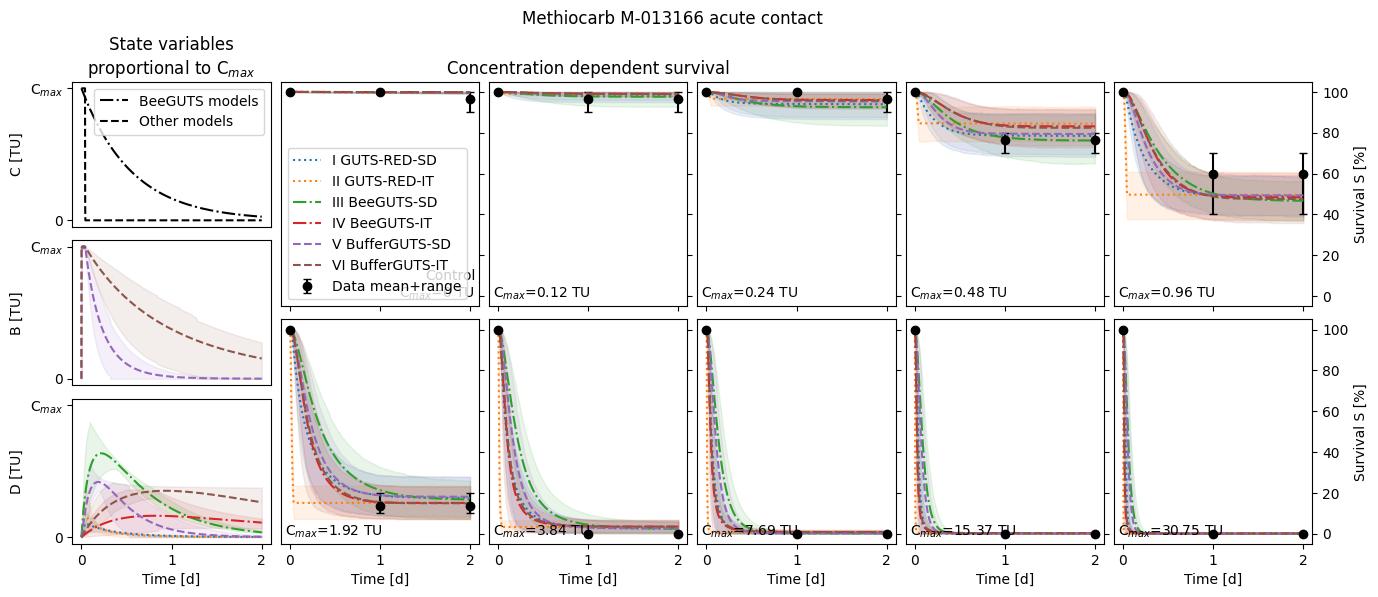

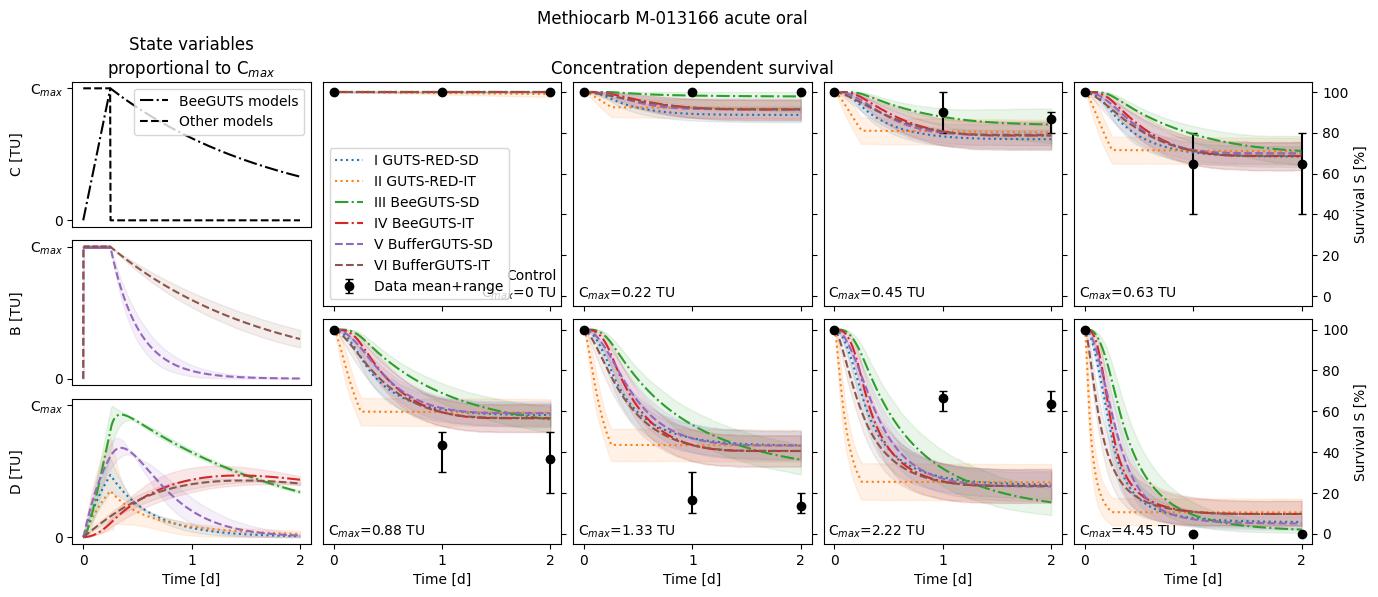

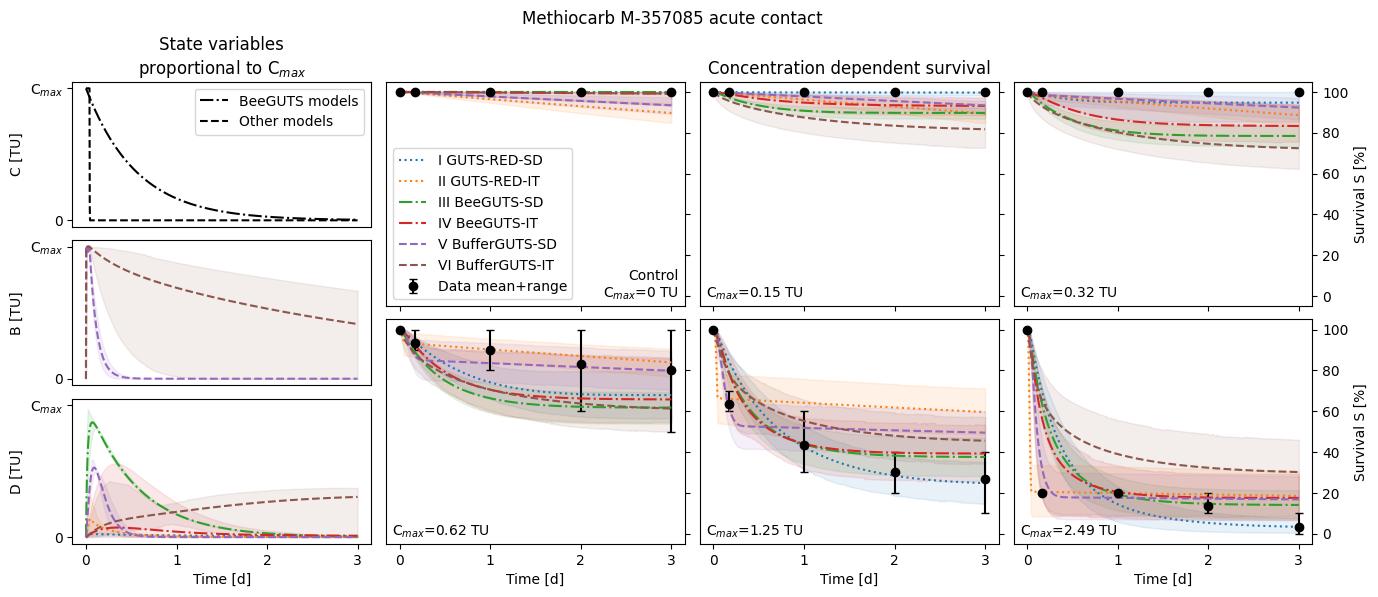

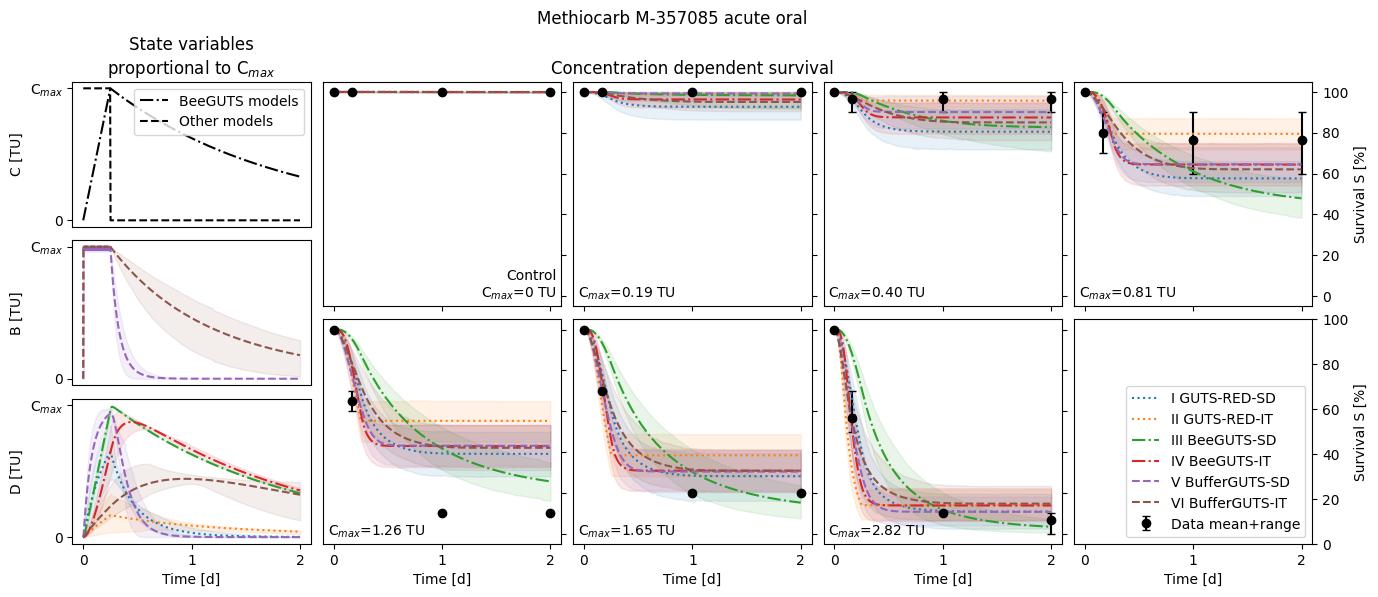

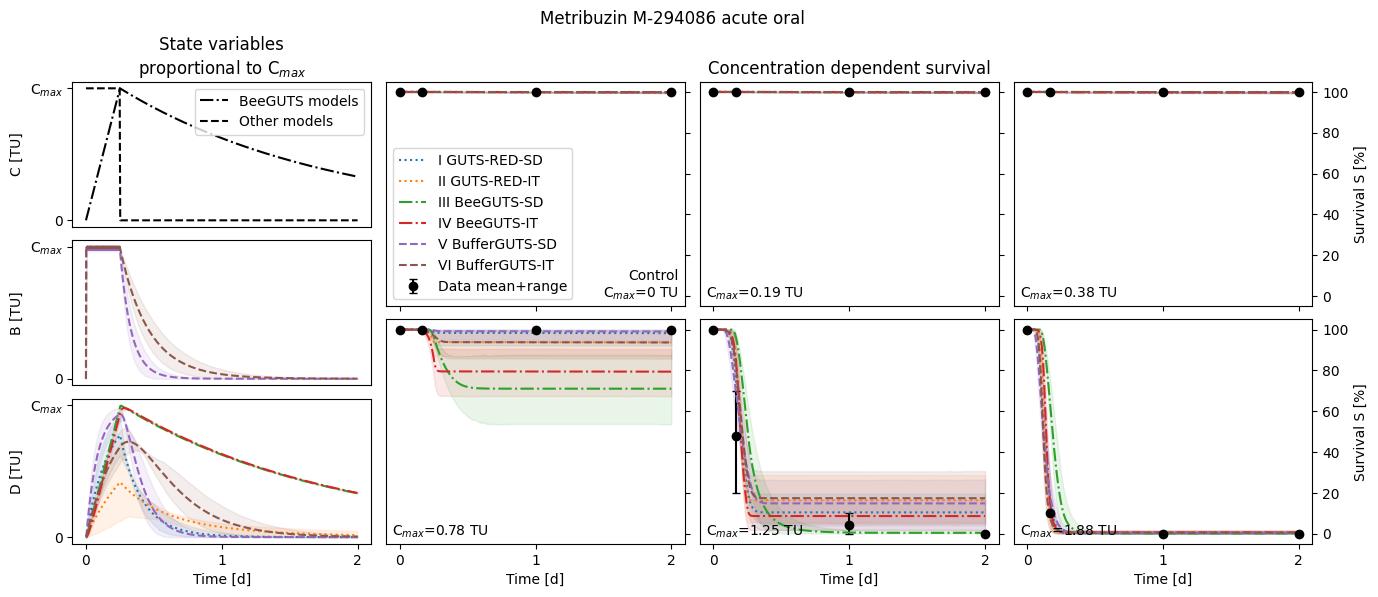

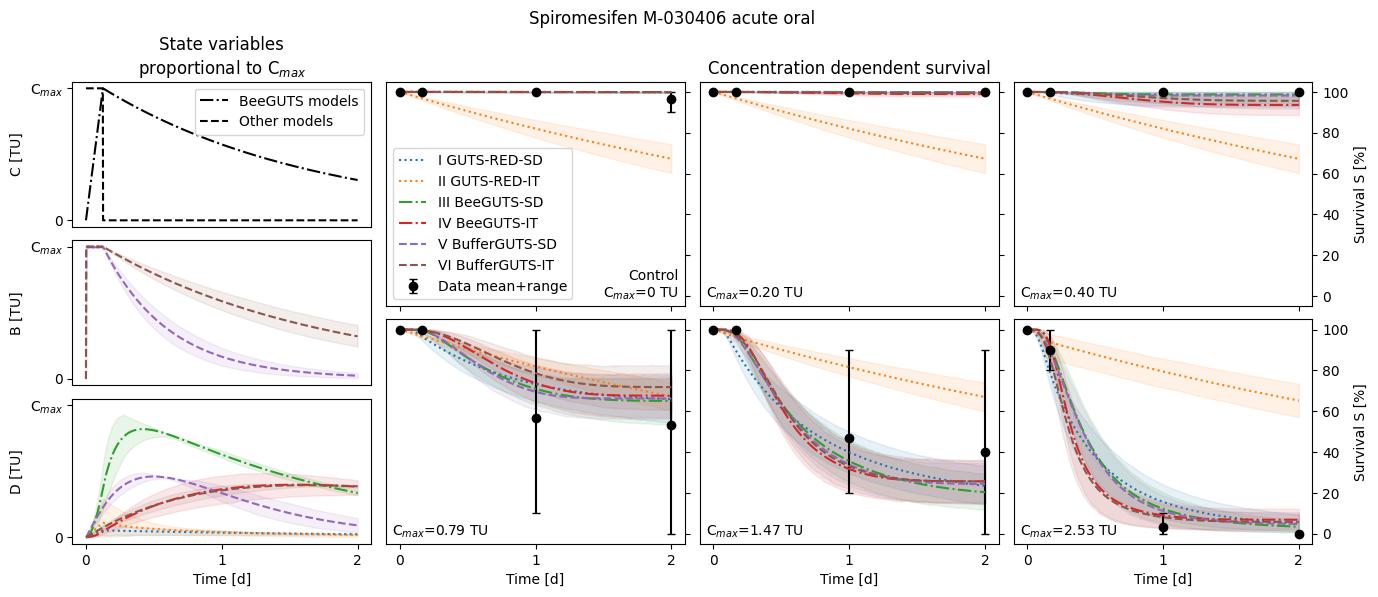

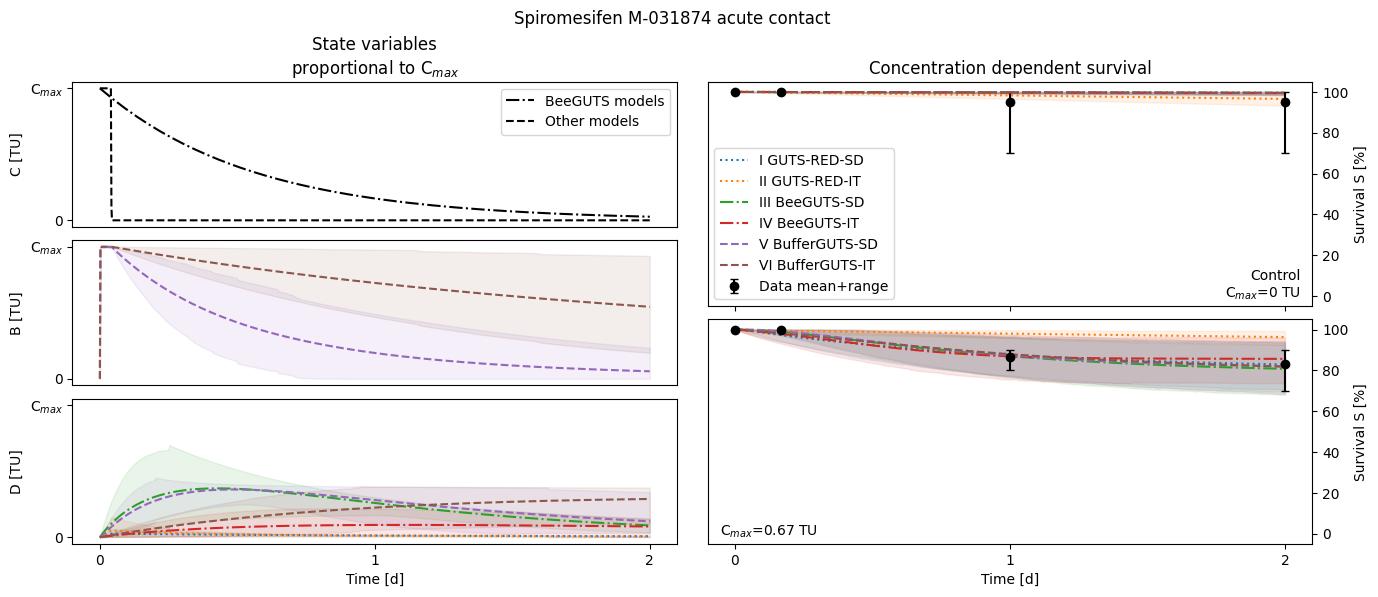

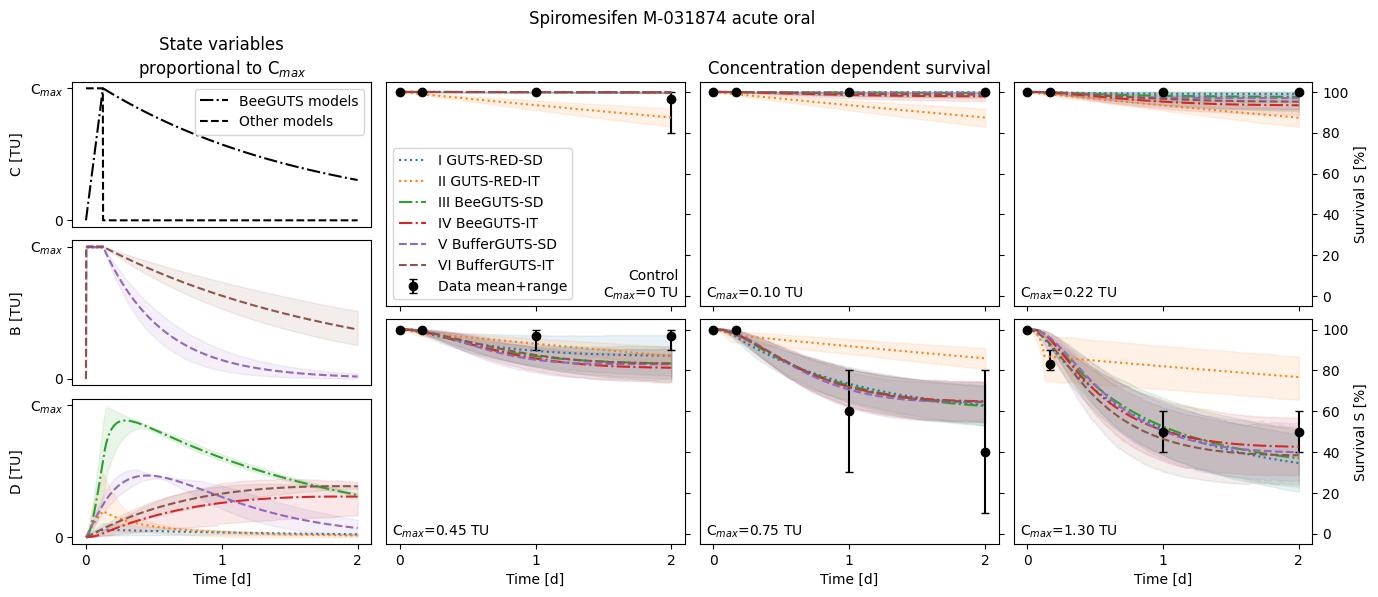

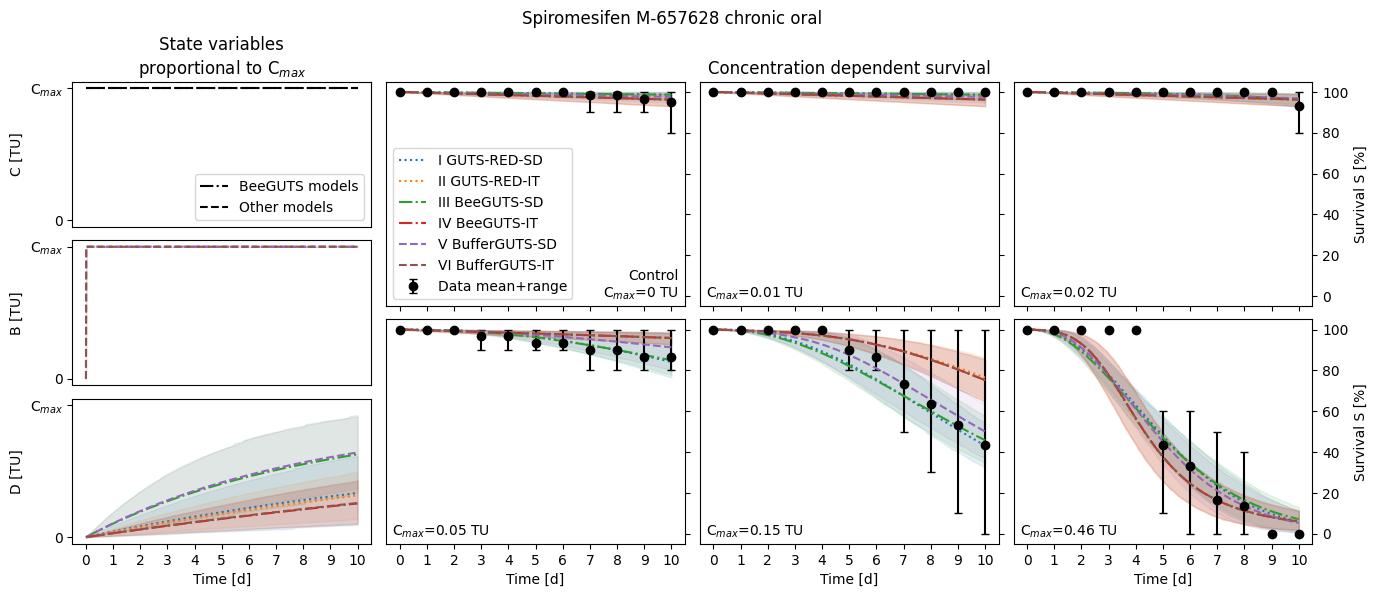

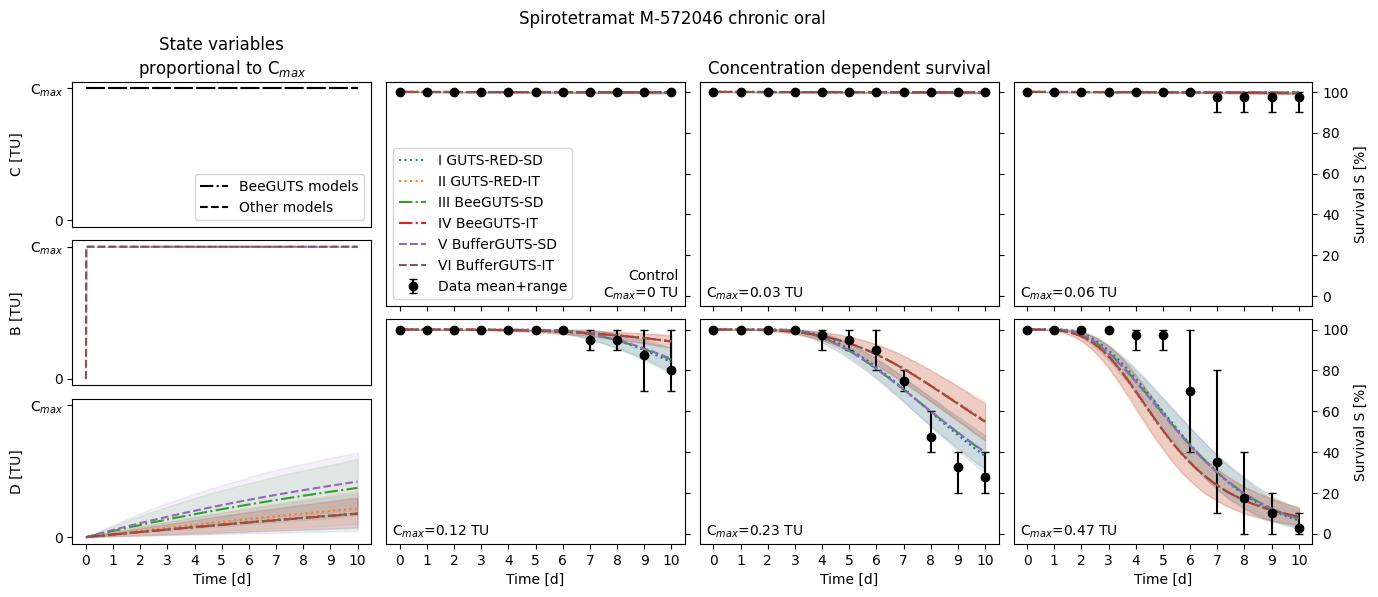

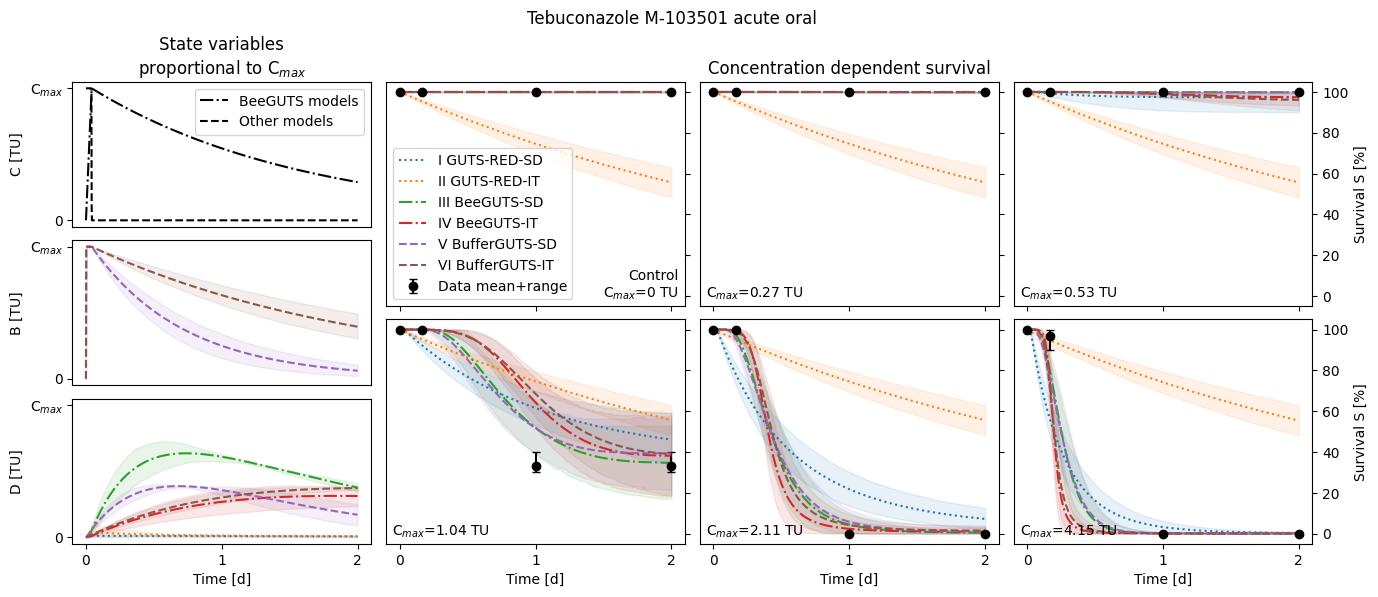

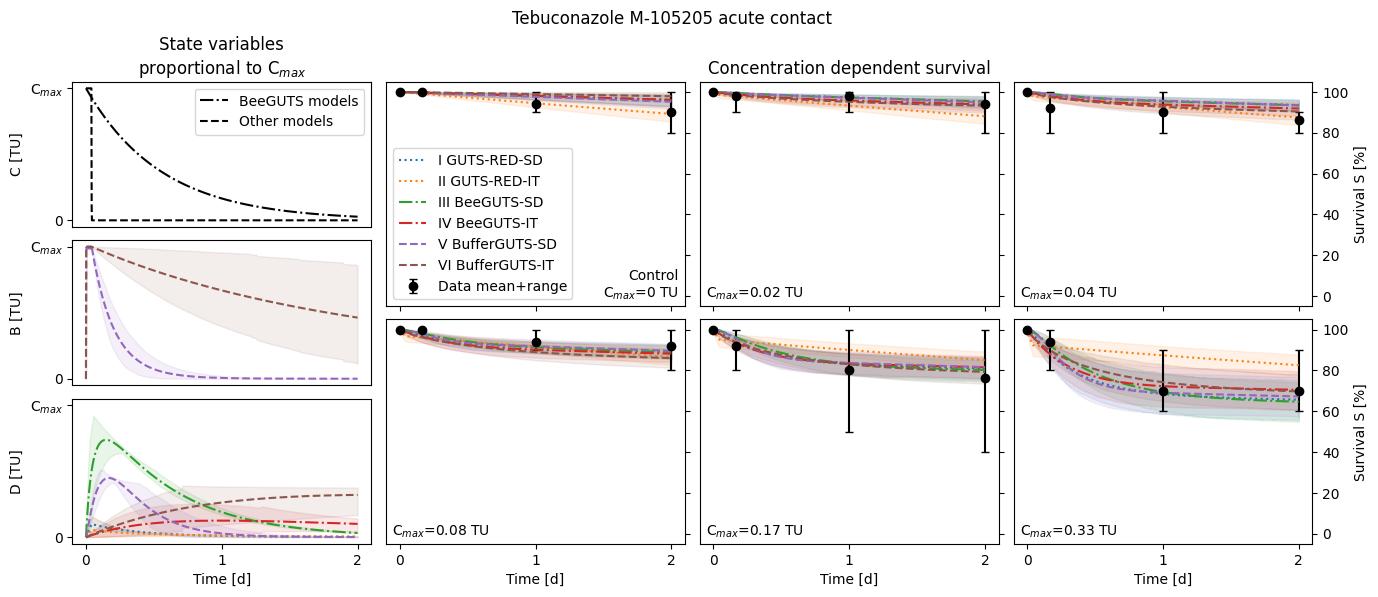

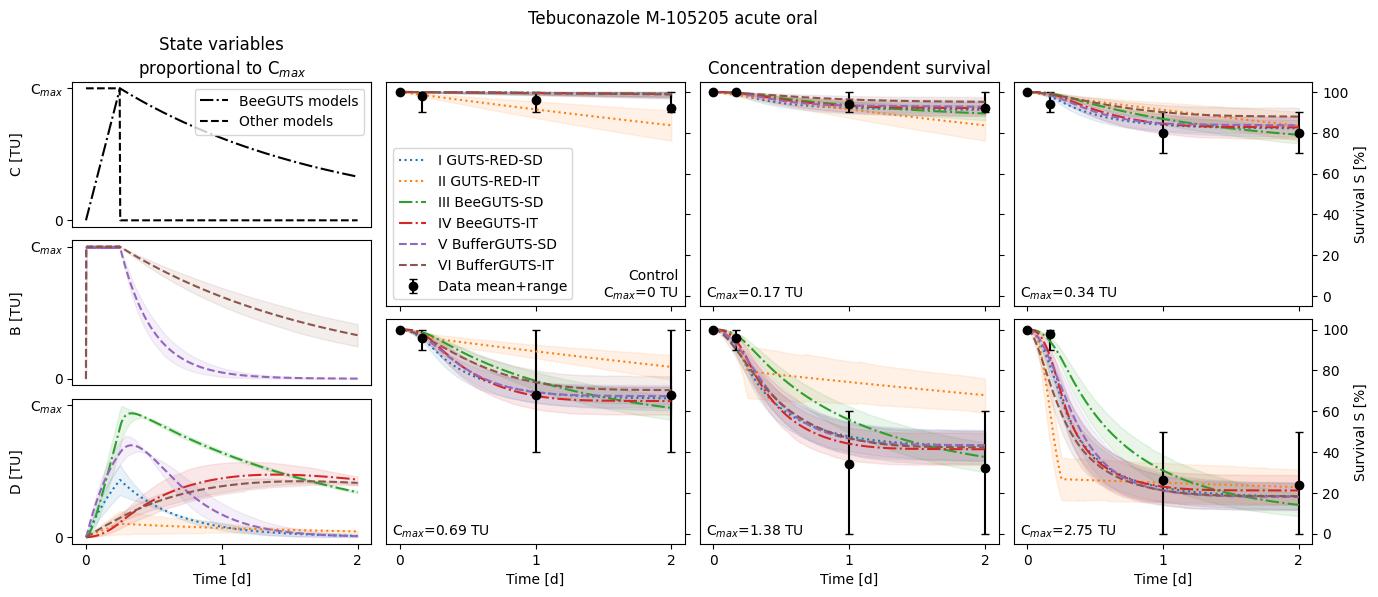

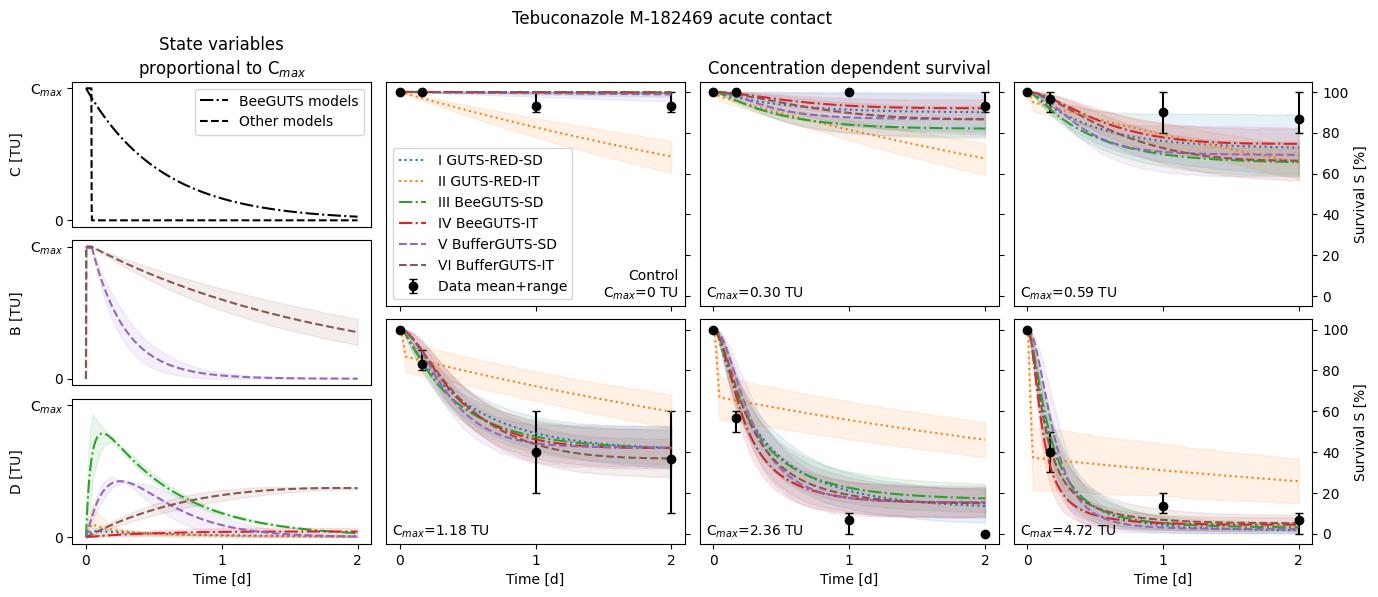

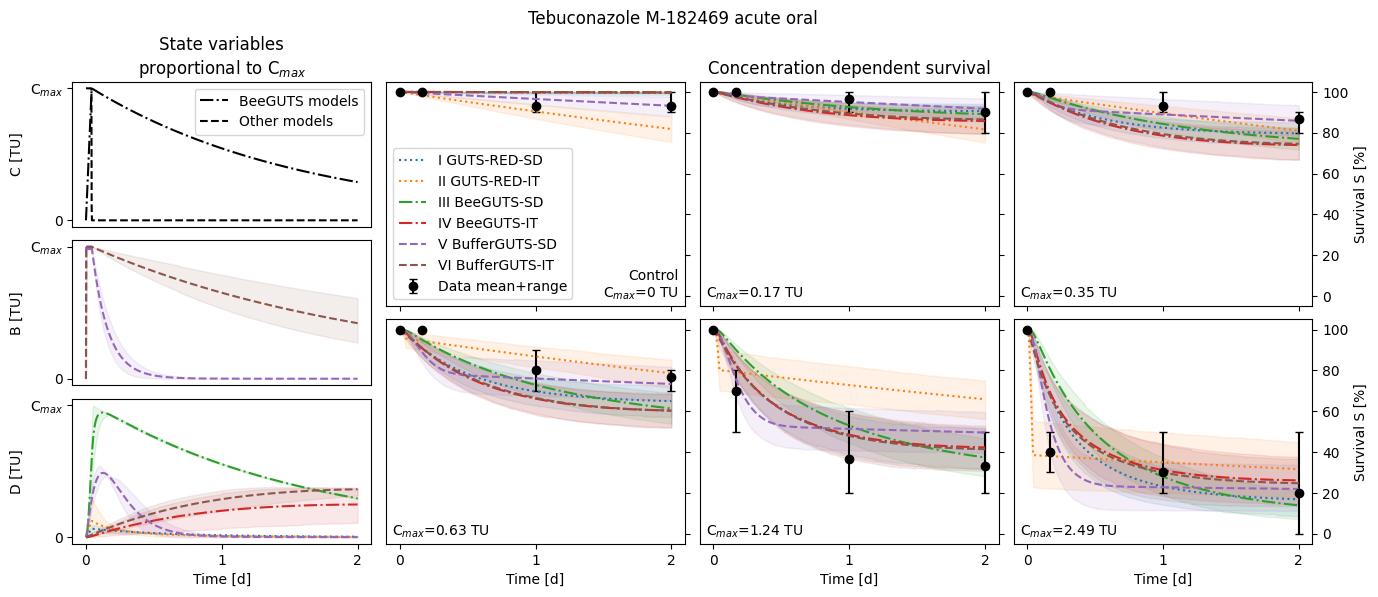

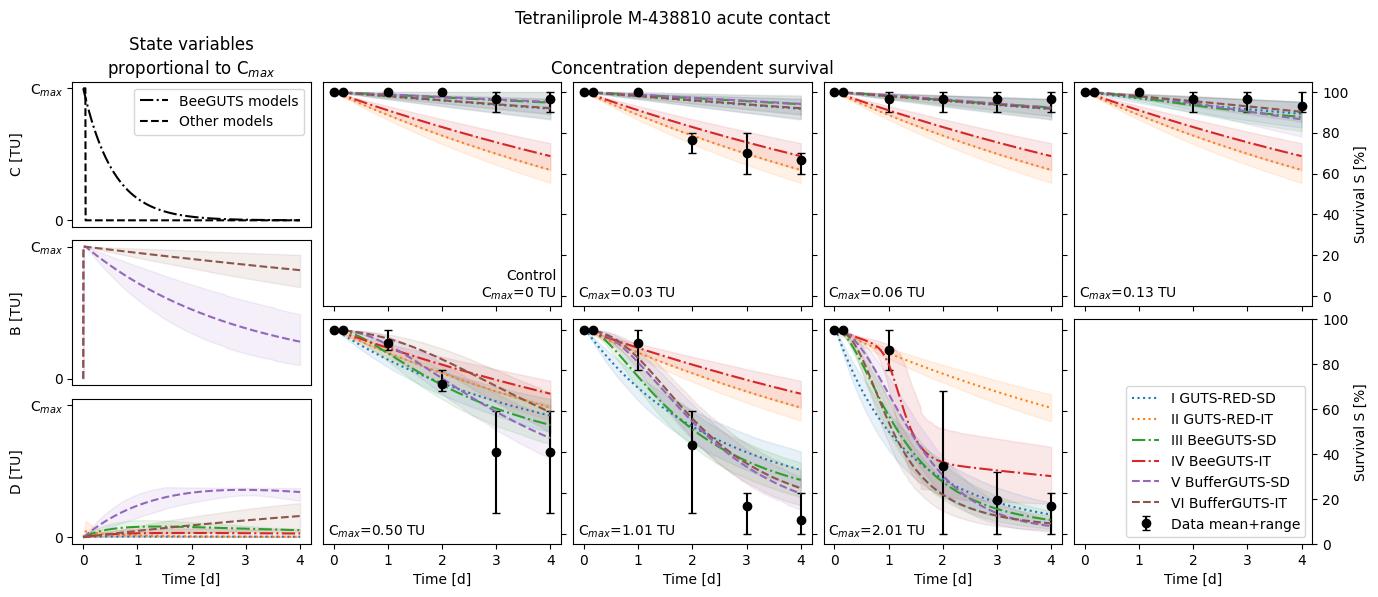

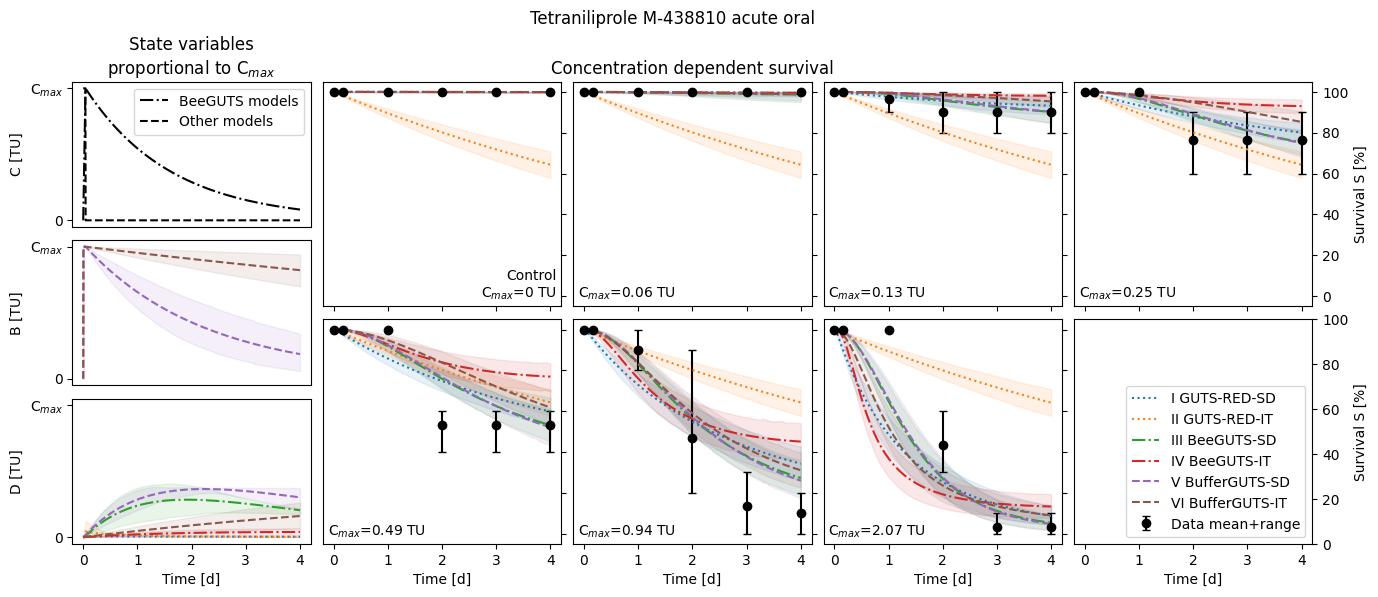

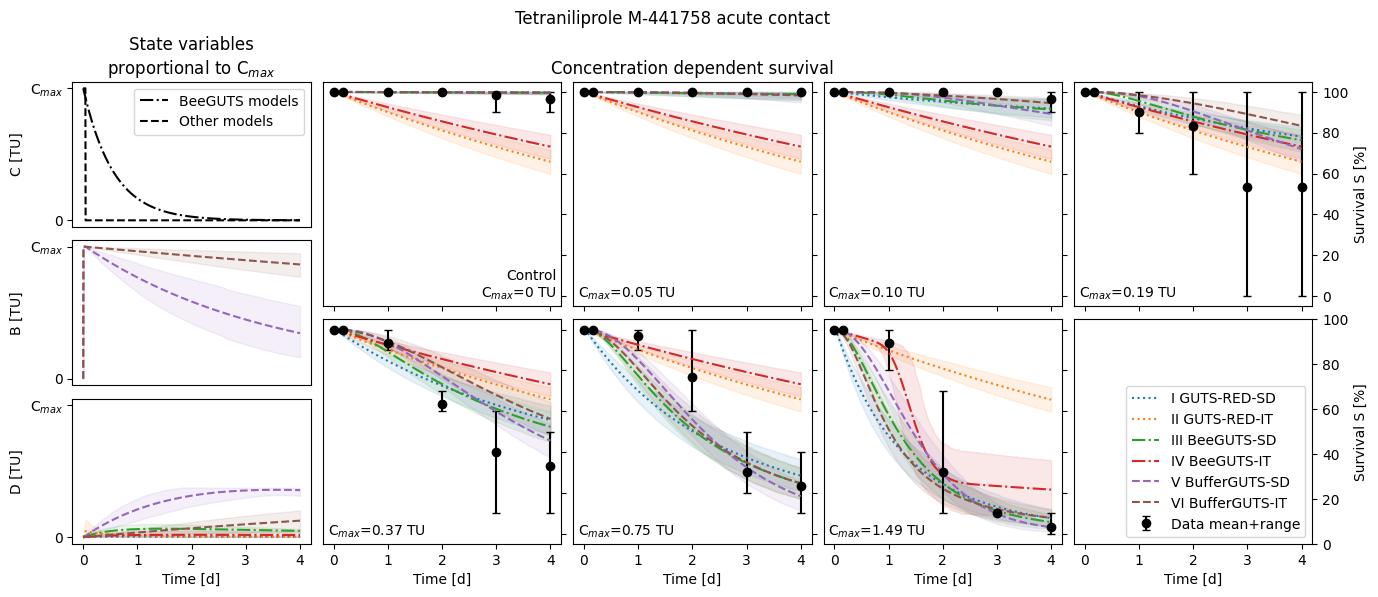

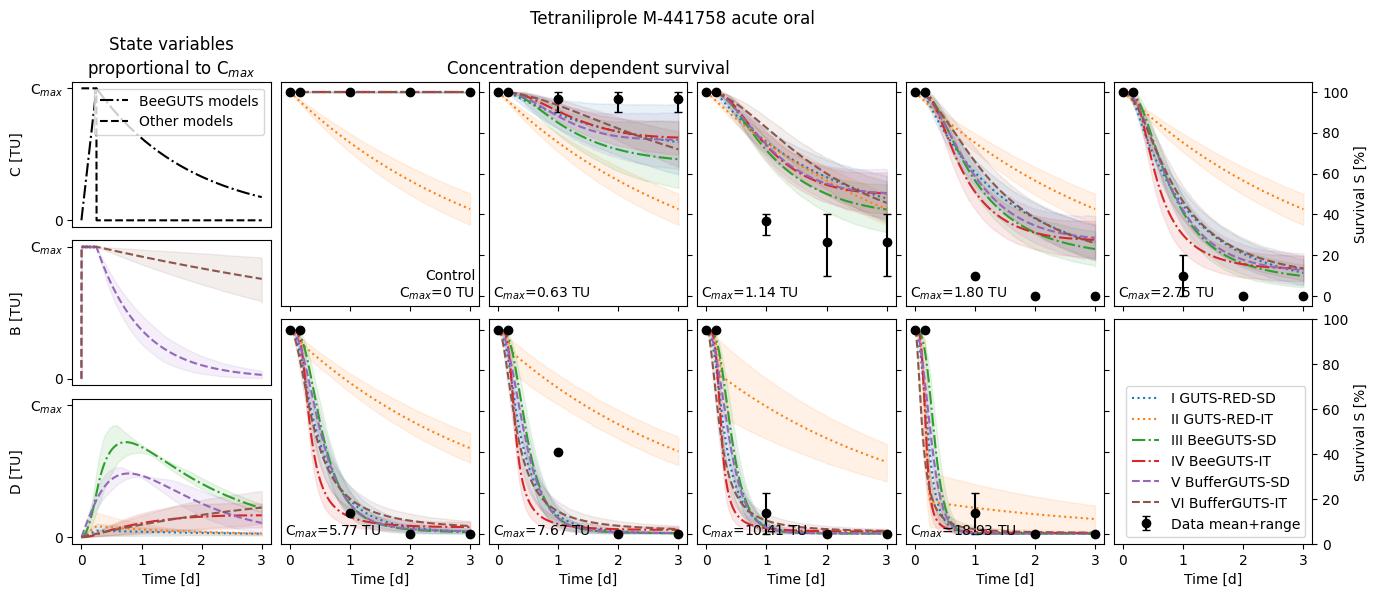

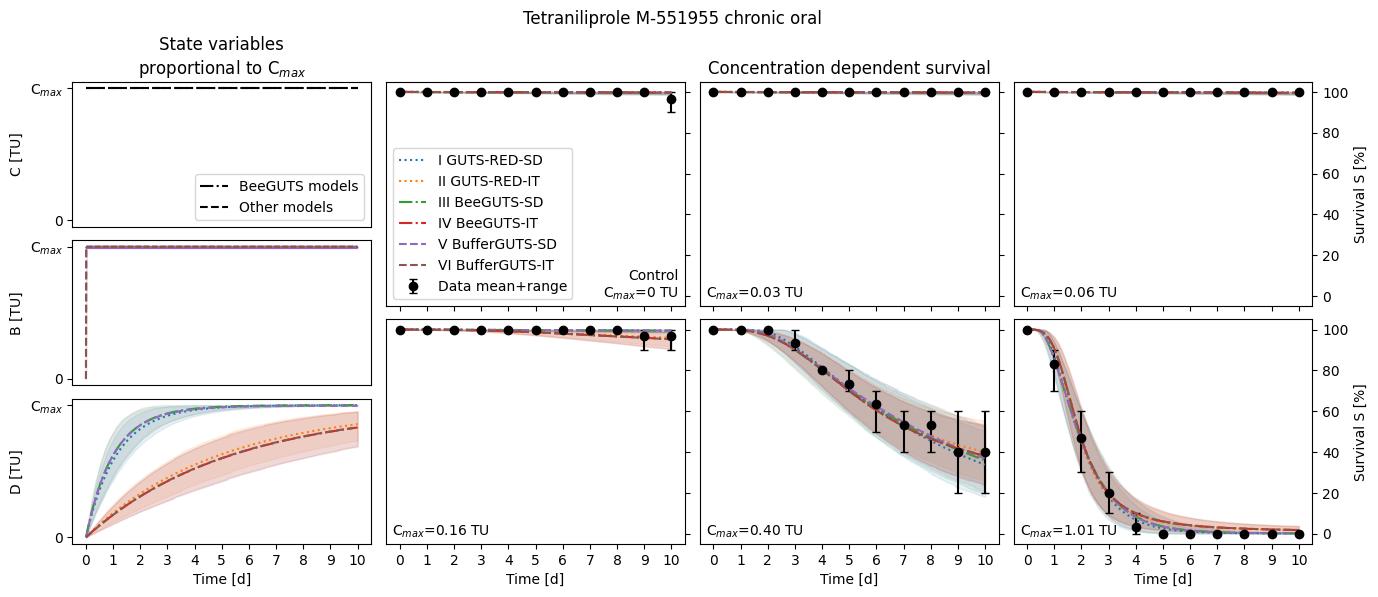

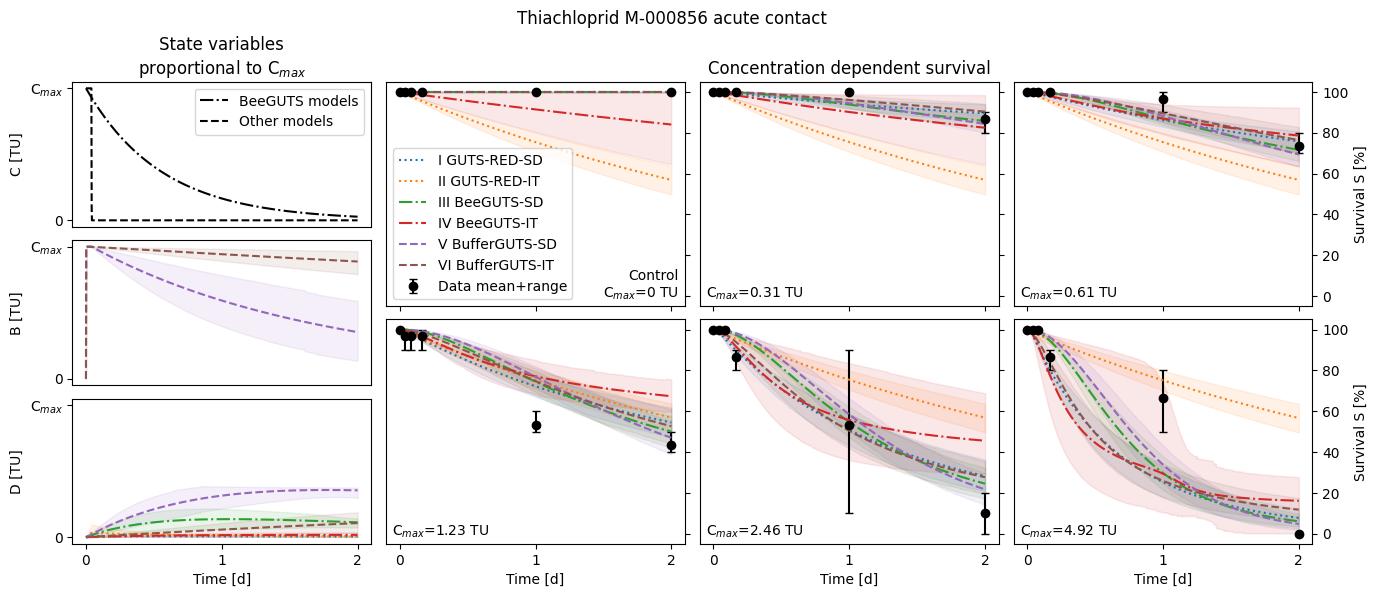

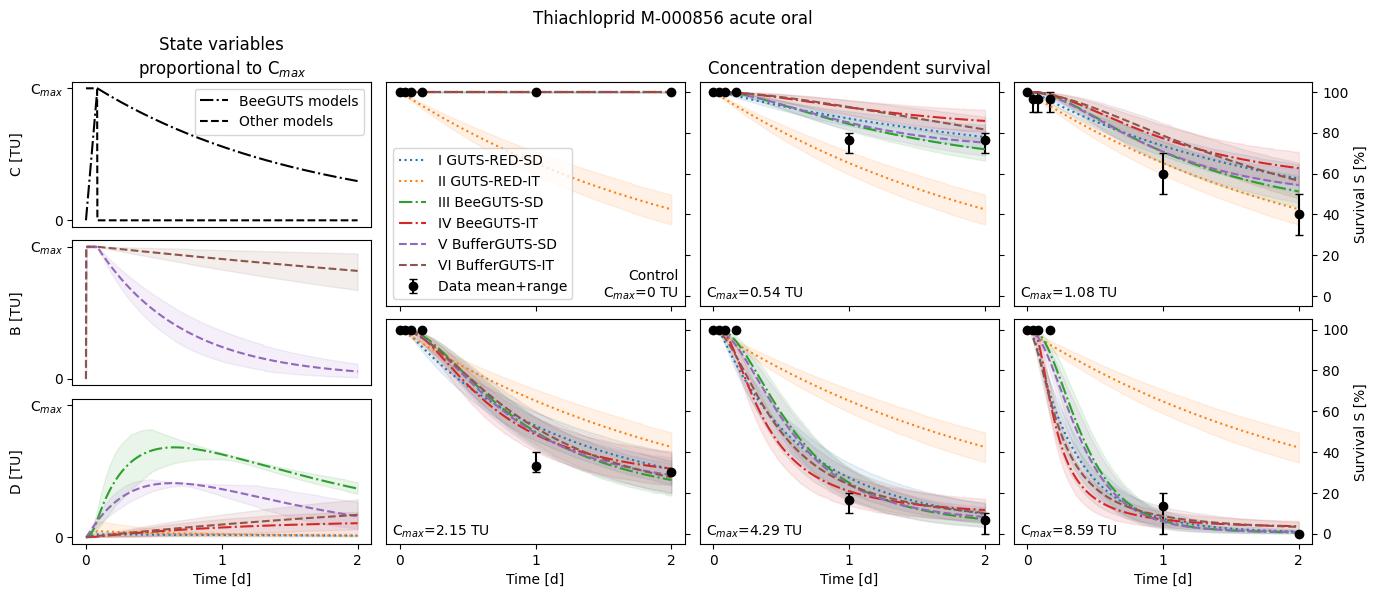

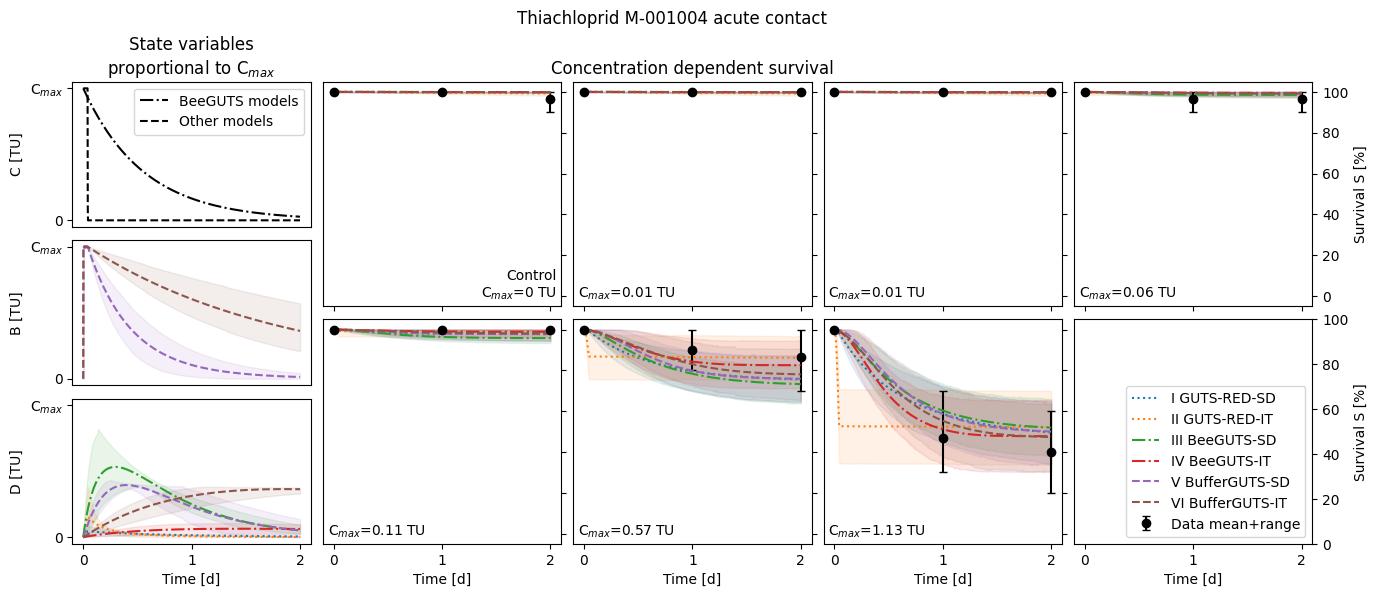

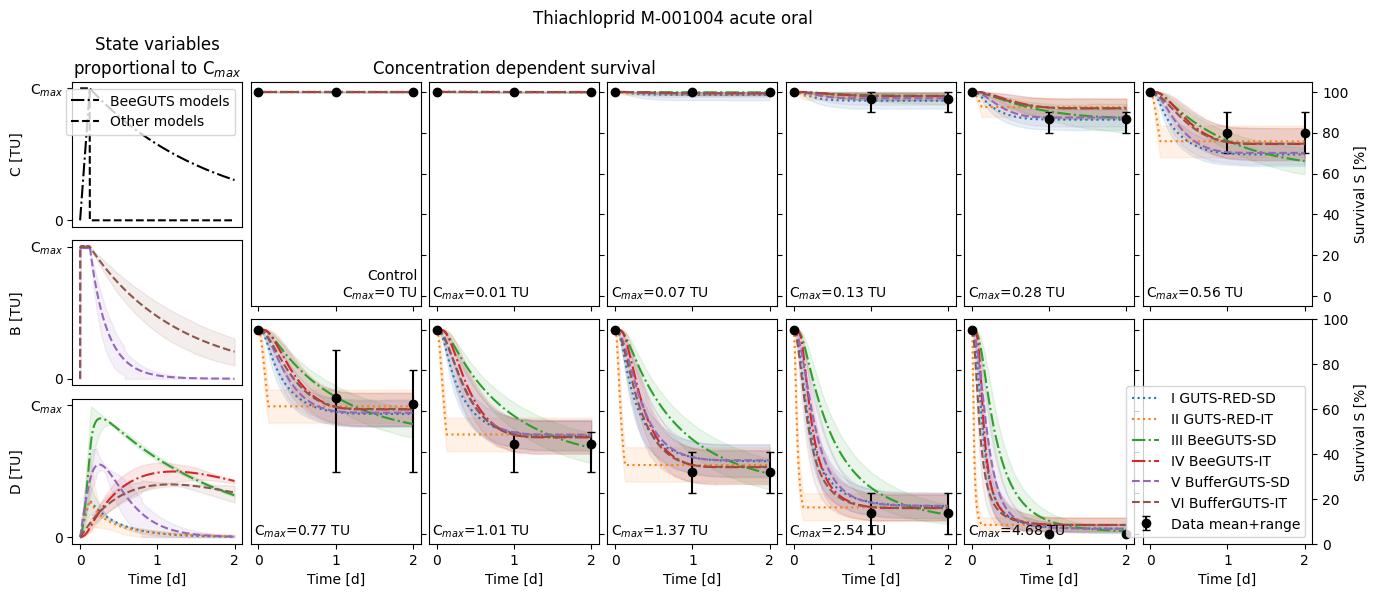

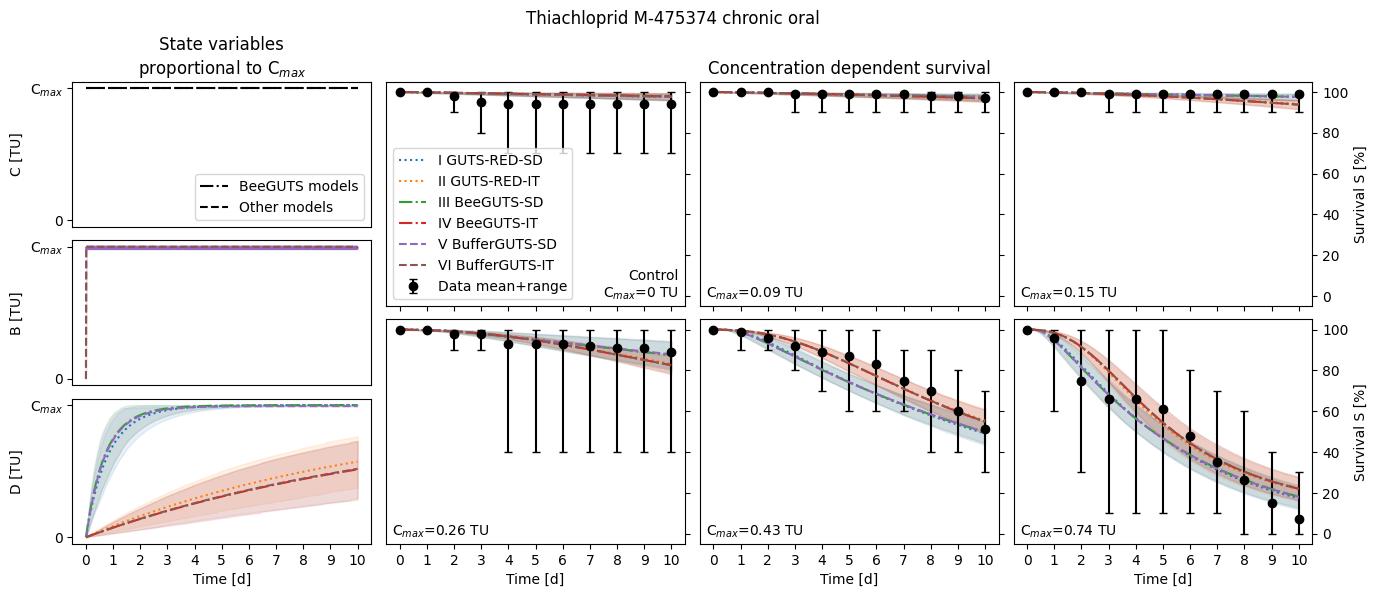


# References for the supporting information

Baas, J., Goussen, B., Miles, M., Preuss, T. G., & Roessink, I. (2022). BeeGUTS-A Toxicokinetic-Toxicodynamic Model for the Interpretation and Integration of Acute and Chronic Honey Bee Tests. *Environmental Toxicology and Chemistry*, *41*(9), 2193–2201. https://doi.org/10.1002/etc.5423

Baudrot, V., & Charles, S. (2021). `morse`: An R-package to analyse toxicity test data. *Journal of Open Source Software*, *6*(68), 3200. https://doi.org/10.21105/joss.03200

EFSA Panel on Plant Protection Products and their Residues (PPR), Ockleford, C., Adriaanse, P., Berny, P., Brock, T., Duquesne, S., Grilli, S., Hernandez-Jerez, A. F., Bennekou, S. H., Klein, M., Kuhl, T., Laskowski, R., Machera, K., Pelkonen, O., Pieper, S., Smith, R. H., Stemmer, M., Sundh, I., Tiktak, A., … Teodorovic, I. (2018). Scientific Opinion on the state of the art of Toxicokinetic/Toxicodynamic (TKTD) effect models for regulatory risk assessment of pesticides for aquatic organisms. *EFSA Journal*, *16*(8), e05377. https://doi.org/10.2903/j.efsa.2018.5377

Jager, T., & Ashauer, R. (2017). *Modelling survival under chemical stress*. Leanpub. https://leanpub.com/guts_book

Uhl, P., Awanbor, O., Schulz, R. S., & Brühl, C. A. (2019). Is Osmia bicornis an adequate regulatory surrogate? Comparing its acute contact sensitivity to Apis mellifera. *PLOS ONE*, *14*(8), e0201081. https://doi.org/10.1371/journal.pone.0201081
